# Peroxisome disruption alters lipid metabolism and potentiates antitumor response with MAPK-targeted therapy in melanoma

**DOI:** 10.1172/JCI166644

**Published:** 2023-10-16

**Authors:** Fan Huang, Feiyang Cai, Michael S. Dahabieh, Kshemaka Gunawardena, Ali Talebi, Jonas Dehairs, Farah El-Turk, Jae Yeon Park, Mengqi Li, Christophe Goncalves, Natascha Gagnon, Jie Su, Judith H. LaPierre, Perrine Gaub, Jean-Sébastien Joyal, John J. Mitchell, Johannes V. Swinnen, Wilson H. Miller, Sonia V. del Rincón

**Affiliations:** 1 Lady Davis Institute,; 2 Department of Experimental Medicine, and; 3 Department of Oncology, McGill University, Montreal, Quebec, Canada.; 4 Laboratory of Lipid Metabolism and Cancer, Department of Oncology, KU Leuven and Leuven Cancer Institute (LKI), Leuven, Belgium.; 5 McGill University Health Centre, Montreal, Quebec, Canada.; 6 Centre Hospitalier Universitaire Sainte Justine, Montreal, Quebec, Canada.; 7 Centre de Recherche, CHU St. Justine, Montréal, Quebec, Canada.

**Keywords:** Oncology, Melanoma

## Abstract

Melanomas reprogram their metabolism to rapidly adapt to therapy-induced stress conditions, allowing them to persist and ultimately develop resistance. We report that a subpopulation of melanoma cells tolerate MAPK pathway inhibitors (MAPKis) through a concerted metabolic reprogramming mediated by peroxisomes and UDP-glucose ceramide glycosyltransferase (UGCG). Compromising peroxisome biogenesis, by repressing PEX3 expression, potentiated the proapoptotic effects of MAPKis via an induction of ceramides, an effect limited by UGCG-mediated ceramide metabolism. Cotargeting PEX3 and UGCG selectively eliminated a subset of metabolically active, drug-tolerant CD36^+^ melanoma persister cells, thereby sensitizing melanoma to MAPKis and delaying resistance. Increased levels of peroxisomal genes and *UGCG* were found in patient-derived MAPKi-relapsed melanomas, and simultaneously inhibiting PEX3 and UGCG restored MAPKi sensitivity in multiple models of therapy resistance. Finally, combination therapy consisting of a newly identified inhibitor of the PEX3-PEX19 interaction, a UGCG inhibitor, and MAPKis demonstrated potent antitumor activity in preclinical melanoma models, thus representing a promising approach for melanoma treatment.

## Graphical abstract

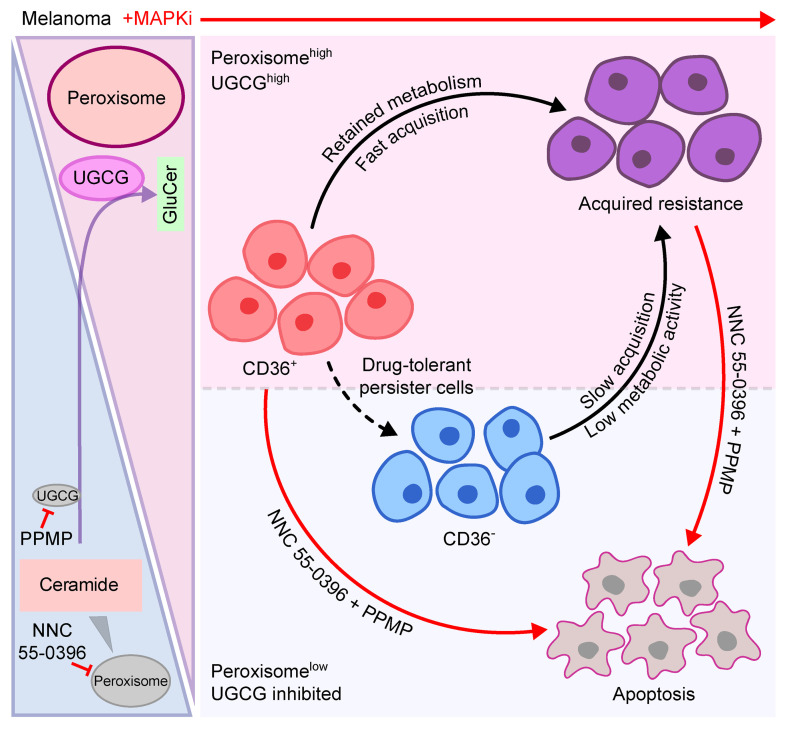

## Introduction

Melanoma is the deadliest form of skin cancer. Current treatments for patients with metastatic melanoma include therapies that target the MAPK pathway (BRAF and MEK inhibitors) and immunotherapies targeting immune checkpoints. However, as with all therapies, MAPK- and immune-targeted treatments have their limitations, often with patients being intrinsically resistant or developing resistance (1). Expanding the toolbox of treatments available for patients with melanoma requires a thorough understanding of the underlying biology of this disease.

Melanomas typically exhibit high intratumoral heterogeneity and phenotype plasticity, which ultimately promote MAPK inhibitor (MAPKi) resistance, as a combined result of clonal evolution (MAPKi-mediated cell selection) and the emergence of phenotypically and metabolically distinct cell states (2, 3). Metabolic reprogramming is a mechanism through which subsets of melanoma cells adapt to microenvironmental cues and MAPK-targeted therapy (4–8). PGC1α-mediated mitochondrial oxidative phosphorylation (OXPHOS) and PPARα-mediated fatty acid oxidation (FAO) are 2 metabolic pathways that have emerged as promising targets to overcome therapy resistance in melanoma (5–8).

Peroxisomes are highly specialized membrane-bound organelles with vital metabolic functions, including β oxidation of very-long-chain fatty acids, biosynthesis of C24-bile acids and ether-phospholipids (EPLs), and reactive oxygen species (ROS) metabolism. In mammalian cells, the peroxins PEX3, PEX16, and PEX19 are required for de novo biogenesis of peroxisomes (9, 10). Patients with peroxisomal disorders often exhibit impaired lipid metabolism (11). Moreover, recent studies suggest that dysregulated sphingolipid metabolism, marked by increased levels of ceramide and altered sphingomyelin abundance, might be a potential biomarker of peroxisomal disorders (12–15), indicating a previously underappreciated role of peroxisomes in sphingolipid metabolism. Moreover, emerging evidence highlights the peroxisome as playing significant roles in maintaining human health, with altered peroxisome functions impacting an expanding list of diseases, including cancer (16). Although there has been a growing appreciation for the roles of EPLs and ceramides in melanoma progression, stress response, and drug sensitivity (17, 18), whether peroxisomes play an essential role in these processes remains largely underexplored.

Herein, we sought to investigate the role of peroxisome-mediated lipid metabolism in melanoma response and resistance to MAPK inhibition. We chose genetic inhibition of *PEX3* as a mode to disrupt peroxisome biogenesis in a panel of melanoma cells harboring different genetic driver mutations. Reduced PEX3 expression in melanoma cells potentiated their response to MAPK inhibition in vitro and in vivo. Using mouse models and lipidomic analysis, we showed that peroxisome-deficient melanoma cells relied on the UDP-glucose ceramide glucosyltransferase–mediated (UGCG-mediated) ceramide metabolism for survival. Interrogating a previously published single-cell RNA-sequencing (scRNA-seq) data set (19), we identified a CD36^+^ cell-state population with dependence on peroxisome/UGCG-mediated metabolic rewiring for MAPKi tolerance. Finally, we identified an inhibitor of PEX3-PEX19 binding and showed that it worked in concert with a UGCG inhibitor to potentiate melanoma response to MAPK-targeted therapy.

## Results

### Compromising peroxisome biogenesis potentiates melanoma cell response to MAPK-targeted therapies.

**Figure 1:**
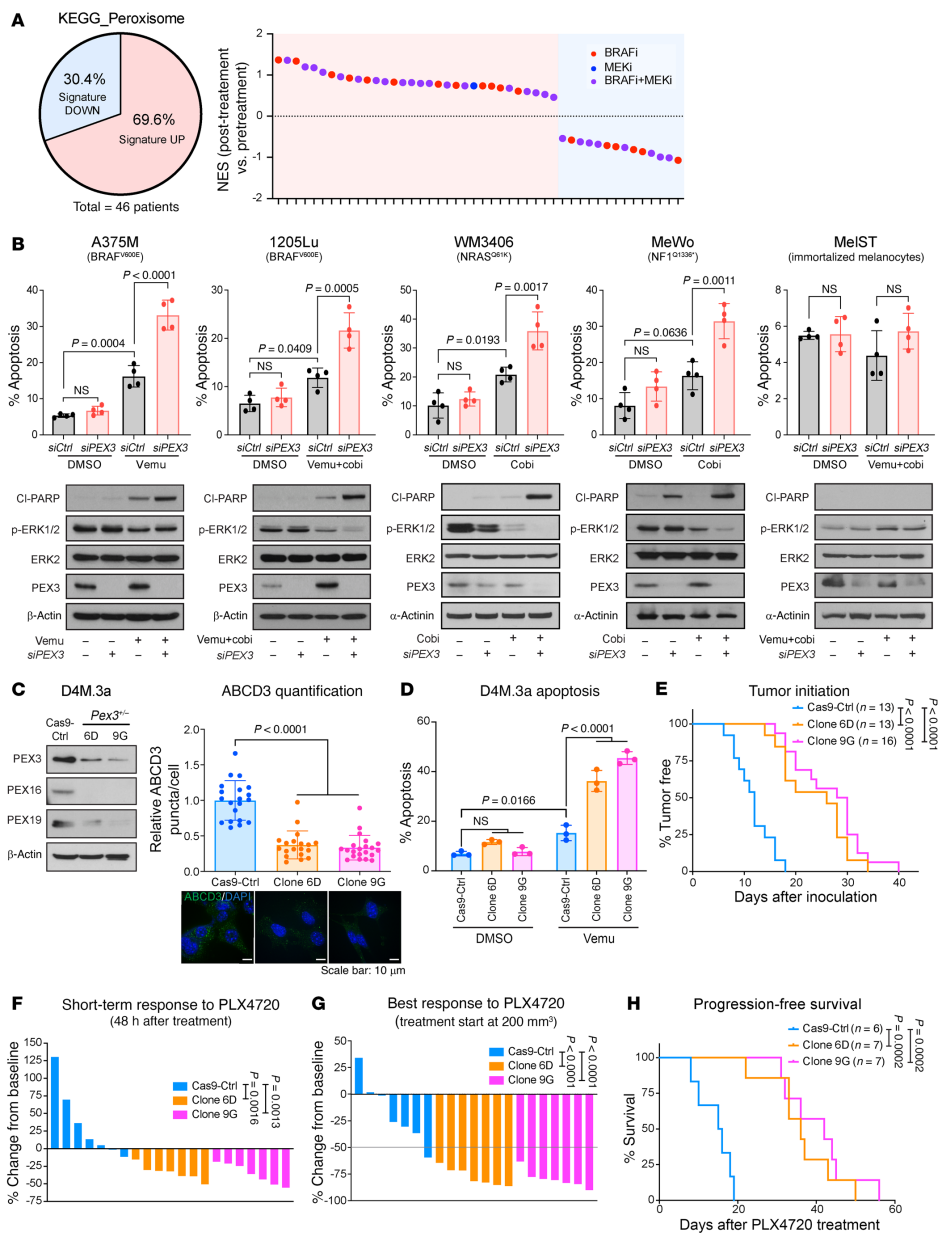
Compromising PEX3 sensitizes melanoma to MAPK inhibition. (**A**) Left: Pie chart showing percentage of patients (*n* = 46) with increased or decreased transcript levels of peroxisome-related genes (KEGG_Peroxisome) after treatment with MAPK-targeted therapies. Right: Normalized enrichment scores (NESs) assessing increase (positive) or decrease (negative) of KEGG_Peroxisome gene set in samples from each patient collected before versus after treatment with indicated MAPK inhibitors. (**B**) Top: Percentage apoptotic cells as measured by the sum of PI/Annexin V double-positive and Annexin V–positive staining. Bottom: Western blot analysis of the indicated proteins in human melanoma cells or immortalized melanocytes (MelST) following *PEX3* knockdown (or *siCtrl* transfection) and treatment with indicated MAPK inhibitors (*n* = 4). Equal volumes of DMSO were added to the control groups. Detailed treatment and timeline are presented in Supplemental Table 1. Two-way ANOVA. Cl-PARP, cleaved poly(ADP-ribose) polymerase. (**C**) Western blot analysis of the indicated proteins (left) and relative number of ABCD3 puncta (right) in D4M.3a Cas9-Ctrl, *Pex3^+/–^* clone 6D, and *Pex3^+/–^* clone 9G cells. Representative immunofluorescent staining for ABCD3 (green) and DAPI nuclear stain (blue) are presented (*n* = 3). Scale bars: 10 μm. One-way ANOVA. (**D**) Percentage apoptosis detected in D4M.3a Cas9-Ctrl, 6D, and 9G cells following vemurafenib (vemu) or DMSO treatment for 24 hours (*n* = 3). Two-way ANOVA. (**E**) Kaplan-Meier curves showing initiation of D4M.3a Cas9-Ctrl–, 6D-, and 9G-derived melanomas. Log-rank test. (**F** and **G**) Waterfall plots showing (**F**) the short-term response (STR, 48-hour treatment) and (**G**) the best response (BR) of D4M.3a Cas9-Ctrl–, 6D-, and 9G-derived melanomas to PLX4720. Values represent percentage volume change of each tumor from baseline. One-way ANOVA. (**H**) Kaplan-Meier curves showing progression-free survival of mice bearing D4M.3a Cas9-Ctrl–, 6D-, and 9G-derived melanomas, fed with PLX4720 chow. Log-rank test. Data in **B**–**D** represent mean ± SD. Number of biological replicates is indicated in each graph.

Continuous exposure to MAPK-targeted therapies, such as BRAF and MEK inhibitors (BRAFis and MEKis), can trigger metabolic reprogramming to promote cell state transitions, known as phenotype plasticity, enabling melanoma cells to persist, ultimately contributing to disease relapse. Given the emerging role of peroxisomes in cancer, we posited that peroxisome biogenesis and lipid metabolism might change in melanoma cells in response to MAPKis. We reanalyzed publicly available RNA-seq data from 4 studies assessing transcriptomic alterations in patient samples collected before and after MAPK-targeted therapy (20–23). Gene set enrichment analysis (GSEA) revealed that approximately 70% of patients showed an overall induction of peroxisome-associated genes after treatment with BRAFis alone or when combined with a MEKi (Figure 1A and Supplemental Figure 1A). Conversely, interrogating the same data, less than 50% of patients showed an enriched gene signature involved in OXPHOS (Supplemental Figure 1A), previously shown to promote MAPKi tolerance in a subset of melanoma cells (5, 24). These in silico–derived data support a potential role of peroxisomes in MAPKi-driven metabolic rewiring, which allows melanoma cells to escape therapeutic pressure.

To formally test the impact of disrupting peroxisome biogenesis on response to MAPKis, we knocked down *PEX3* with siRNAs in 4 human melanoma cell lines harboring different genetic mutations (Figure 1B) to assess whether this would alter their response to MAPK inhibition. Knockdown of *PEX3* significantly decreased the number of peroxisomes in all 4 melanoma cell lines (Supplemental Figure 1B), with minimal impact on cell viability (Figure 1B and Supplemental Figure 1C). We treated BRAF^V600E^-mutant melanoma cell lines with the BRAFi vemurafenib (vemu) alone or in combination with the MEKi cobimetinib (cobi). The non-BRAF-mutant WM3406 and MeWo cells were treated with cobi alone, since the BRAFi would be ineffective in these melanoma subtypes. *PEX3* knockdown sensitized all melanoma subtypes to MAPKi-induced apoptosis, compared with the same cells transfected with scrambled siRNA control (*siCtrl*) (Figure 1B and Supplemental Figure 1C). Similar phenotypes were observed with *PEX19* knockdown (Supplemental Figure 1D). Knockdown of *PEX3* alone, or treatment of *PEX3* knockdowns with vemu+cobi did not induce apoptosis in the nonmalignant melanocyte line termed MelST (Figure 1B) (25).

To model our in vitro results in mice, we sought to knock out *Pex3* in the BRAF^V600E^-mutant melanoma cell line D4M.3a (26). Although none of the 80 clones that we screened showed complete loss of PEX3 expression, we successfully isolated 2 clones (6D and 9G) with single-allele *Pex3* knockout. As expected, characterization of these *Pex3*^+/–^ cell lines showed decreased levels of PEX3, PEX16, and PEX19, and reduced numbers of peroxisomes (Figure 1C). Similar to our human melanoma cell data (Figure 1B), the D4M.3a *Pex3^+/–^* clones were more susceptible to MAPKi-mediated cell death than their Cas9-Ctrl counterparts (Figure 1D). To test the sensitivity of *Pex3^+/–^* melanomas to BRAF inhibition in vivo, we next injected D4M.3a Cas9-Ctrl, *Pex3^+/–^* clone 6D, or *Pex3^+/–^* clone 9G cells into syngeneic mice. A significant delay of tumor onset was observed in mice injected with clone 6D or 9G, compared with mice injected with the Cas9-Ctrl cells (Figure 1E), indicating that compromised peroxisome biogenesis impairs the tumor-initiating ability of melanoma cells. Once initiated, the *Pex3^+/–^* melanomas grew at a similar rate to that of the Cas9-Ctrl group (Supplemental Figure 1E). When melanomas of different genotypes reached a volume of approximately 200 mm^3^, mice were administered a diet containing the vemu analog PLX4720 (27). Mice from the 6D and 9G arms showed a rapid decrease in tumor volume within 48 hours of PLX4720 administration (short-term response, STR), while the Cas9-Ctrl tumors continued increasing in size during this timeframe (Figure 1F). As the study continued, 6D and 9G treatment groups showed a significantly improved best response (BR) in tumor shrinkage and improved progression-free survival (PFS), compared with the Cas9-Ctrl treatment group (Figure 1, G and H). The increased treatment benefit in mice bearing *Pex3^+/–^* melanomas was recapitulated in a separate cohort of mice that were administered PLX4720 chow when their tumors reached 800 mm^3^ in size (Supplemental Figure 1, F–H).

### Lipidomic analyses of Pex3^+/–^ D4M.3a melanoma cells reveal altered levels of ceramide-derived lipid species and increased metabolic vulnerability.

Next, we investigated potential mechanisms through which inhibiting peroxisomes improves MAPKi response in melanoma. Our data suggested that compromised peroxisome biogenesis potentiates MAPKi-induced apoptosis through a mechanism largely independent of alterations in ROS homeostasis and mitochondrial respiration (Supplemental Figure 2, A–G).

**Figure 2:**
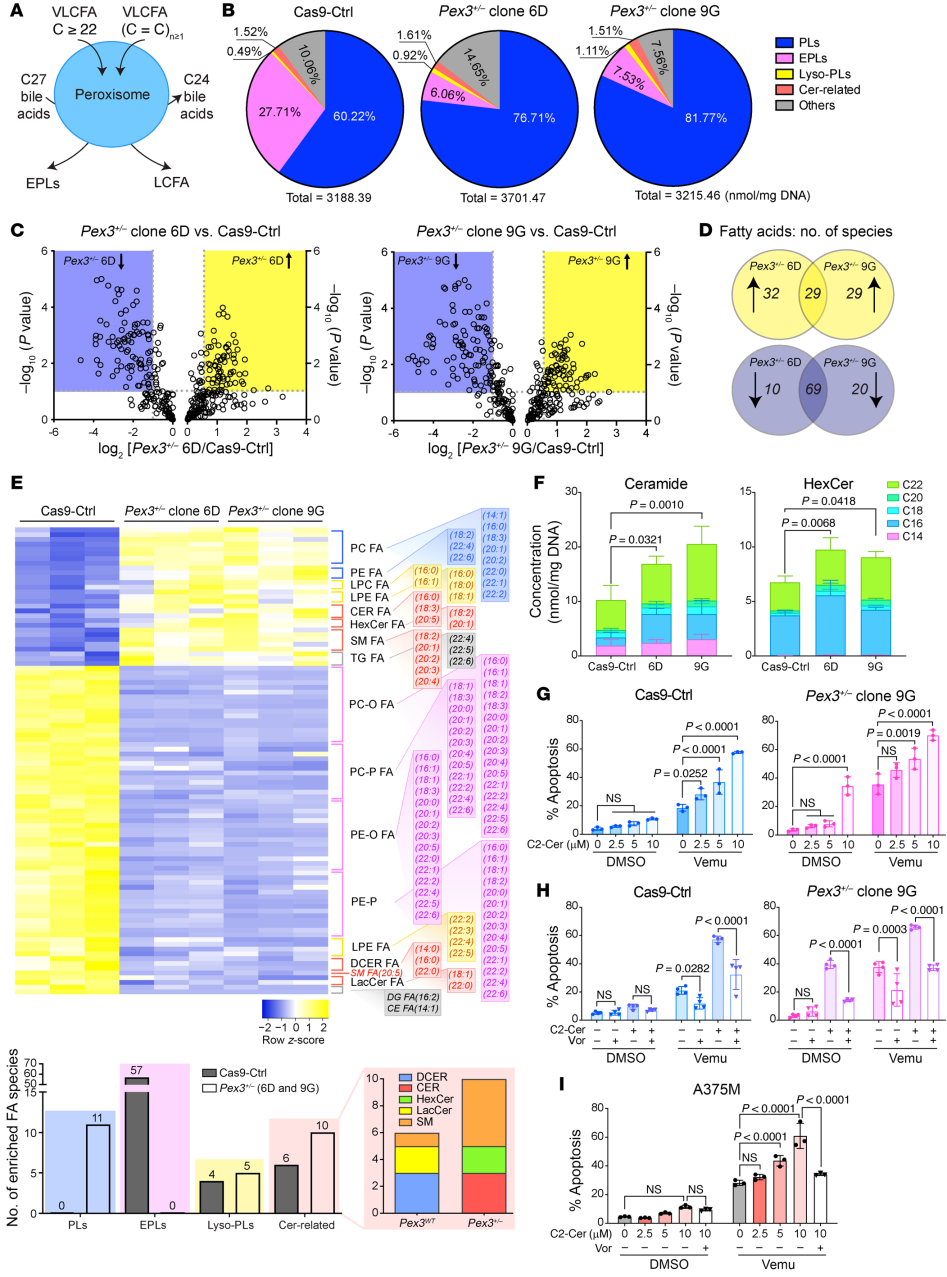
*Pex3^+/–^* D4M.3a melanoma cells have altered lipidomes. (**A**) Schematic of peroxisome-mediated lipid metabolism. VLCFA, very-long-chain fatty acids. (**B**) Pie charts showing lipid composition (relative abundance of each lipid family in percentage total) in D4M.3a Cas9-Ctrl, *Pex3^+/–^* clone 6D, and *Pex3^+/–^* clone 9G cells. Concentrations of each lipid family (normalized to mg DNA) are indicated (*n* = 3). (**C**) Volcano plots comparing abundance of lipid species in clone 6D versus Cas9-Ctrl (left) and clone 9G versus Cas9-Ctrl (right). Yellow and blue shades highlight respective increased (≥1.5-fold) and decreased (≤1.5-fold) lipid species in *Pex3^+/–^* cells relative to Cas9-Ctrl D4M.3a cells. (**D**) Venn diagrams showing lipid species that were significantly increased (top) or decreased (bottom) in D4M.3a clone 6D and clone 9G cells, compared with Cas9-Ctrl cells. (**E**) Top: Heatmap showing lipid species that were commonly altered in D4M.3a *Pex3^+/–^* (6D and 9G) cells compared with Cas9-Ctrl cells. Bottom: Number of lipid species, categorized by family, enriched in D4M.3a Cas9-Ctrl or *Pex3^+/–^* (6D and 9G) cells. PC, phosphatidylcholine; PE, phosphatidylethanolamine; LPC, lysophosphatidylcholine; LPE, lysophosphatidylethanolamine; TG, triglyceride; PE-O, 1-alkyl,2-acylphosphatidylethanolamine; PC-O, 1-alkyl,2-acylphosphatidylcholine; PC-P, 1-alkenyl,2-acylphosphatidylcholine; PE-P, 1-alkenyl,2-acylphosphatidylethanolamine; DG, diacylglyceride; CE, cholesterol ester. (**F**) Concentrations of ceramides (left) and hexosylceramides (HexCer, right) detected in D4M.3a Cas9-Ctrl, 6D, and 9G cells (*n* = 3). Two-way ANOVA. (**G**–**I**) Percentage apoptosis (PI^+^/Annexin V^+^, PI^–^/Annexin V^+^) detected in DMSO- or vemu-treated (**G** and **H**) D4M.3a Cas9-Ctrl (left) or *Pex3^+/–^* clone 9G (right) or (**I**) A375M cells. Cells were pretreated with (**G** and **I**) C2-ceramide (C2-Cer) at escalated doses or with (**H** and **I**) C2-Cer (10 μM) and vorinostat (Vor, 1 μM) 24 hours prior to vemu treatment (*n* = 4 for **H**, *n* = 3 for **G**, **I**). Two-way ANOVA. All data represent mean ± SD.

Given the essential role of peroxisomes in cellular lipid metabolism (Figure 2A), we performed lipidomic analyses using our stable *Pex3^+/–^* D4M.3a (6D and 9G) models to assess whether alterations in lipid species underpin the antitumor phenotypes observed when peroxisome biogenesis is repressed. Our data revealed that *Pex3^+/–^* D4M.3a cells have an increased abundance of phospholipids (PLs), dramatically decreased levels of EPLs, and a slight increase in lysophospholipids (Lyso-PLs) (Figure 2B). We next compared the abundance of each fatty acid–containing species in *Pex3^+/–^* cells versus Cas9-Ctrl cells. We found 29 commonly upregulated and 69 commonly downregulated species in *Pex3^+/–^* D4M.3a cells (Figure 2, D and E). Similar to the observed changes in their relative concentrations (Figure 2B), 11 PL species were increased and 57 EPL species were decreased in the *Pex3^+/–^* cells compared with the Cas9-Ctrl cells (Figure 2E). The increased PL and decreased EPL levels in *Pex3^+/–^* D4M.3a cells are consistent with clinical phenotypes detected in patients with peroxisomal disorders and corresponding *PEX3* biallelic mutant cell lines in vitro (28–30). The latter support the robustness of our lipidomic analyses and the *Pex3^+/–^* D4M.3a cells as a reliable model to understand the biological implications of altering peroxisome biogenesis.

Interestingly, our lipidomic analysis revealed changes in several sphingolipid species, centering on the synthesis and metabolism of ceramides (Figure 2, E and F, and Supplemental Figure 3, A and B). We observed increased levels of ceramides and hexosylceramides (HexCers) (Figure 2, E and F), and decreased levels of dihydroceramides (DCERs) and lactosylceramides (LacCers) (Supplemental Figure 3A). Notably, although several sphingomyelin (SM) species were altered in *Pex3^+/–^* cells (Figure 2E), no consistent changes were observed in total SM level (Supplemental Figure 3A). Our data are consistent with previous studies showing elevated ceramide levels and altered composition of SM species in patients with peroxisomal disorders (13, 14), further supporting a role of peroxisomes as regulators of sphingolipid metabolism (15). Intriguingly, myriocin, an inhibitor of de novo sphingolipid synthesis, blocked the sensitization of *Pex3^+/–^* cells to vemu-induced apoptosis (Supplemental Figure 3, B and C). These data indicate that the hypersensitivity of *Pex3^+/–^* cells to vemu is likely attributable to increased levels of certain sphingolipid species, namely ceramides and HexCers, depletion of both of which was confirmed after myriocin treatment (Supplemental Figure 3C, bottom). Knockdown of *PEX3* or *PEX19* in A375M cells also increased the levels of ceramides and HexCers (Supplemental Figure 3D).

Ceramides are critical mediators of cell fate and lie at the nexus of sphingolipid metabolism (Supplemental Figure 3B). While increased ceramides can compensate for the loss of EPLs due to disrupted peroxisomal function (15), several cell stressors stimulate the production of ceramides to promote apoptosis (31, 32). We hypothesized that the increased susceptibility of peroxisome-deficient melanoma cells to MAPKi-induced apoptosis is mediated via a mechanism involving ceramide-dependent cell death. To test this, we used C2-ceramide (C2-Cer), a cell-permeable ceramide analog. While C2-Cer demonstrated limited cytotoxicity in Cas9-Ctrl D4M.3a cells, it potentiated vemu-induced apoptosis in a dose-dependent manner (Figure 2G). In *Pex3^+/–^* (9G) cells, which are characterized by increased ceramide levels (Figure 2F), C2-Cer alone was able to induce cell death, albeit at a higher dose (Figure 2G), suggesting a potential metabolic vulnerability in peroxisome-compromised cells. When combined with vemu, C2-Cer further induced apoptosis in *Pex3^+/–^* 9G cells (Figure 2G). Next, we used the HDAC inhibitor vorinostat (Vor), known to induce peroxisome biogenesis (33, 34), to test whether an increase in peroxisomes could protect cells from ceramide/vemu-induced apoptosis. As expected, Vor increased peroxisome numbers in both Cas9-Ctrl and *Pex3^+/–^* 9G cells (Supplemental Figure 3E) and significantly decreased apoptosis induced by C2-Cer and vemu (Figure 2H). Notably, induction of peroxisomes protected *Pex3^+/–^* 9G cells against high-dose C2-Cer (Figure 2H), further supporting a role of peroxisomes in ceramide metabolism. Similar results were observed in A375M cells (Figure 2I and Supplemental Figure 3F). Together, our data suggest that reducing peroxisomes in melanoma cells potentiates their response to MAPKis through increased ceramides.

### Dual inhibition of peroxisome biogenesis and UGCG potentiates melanoma response to MAPKis.

**Figure 3:**
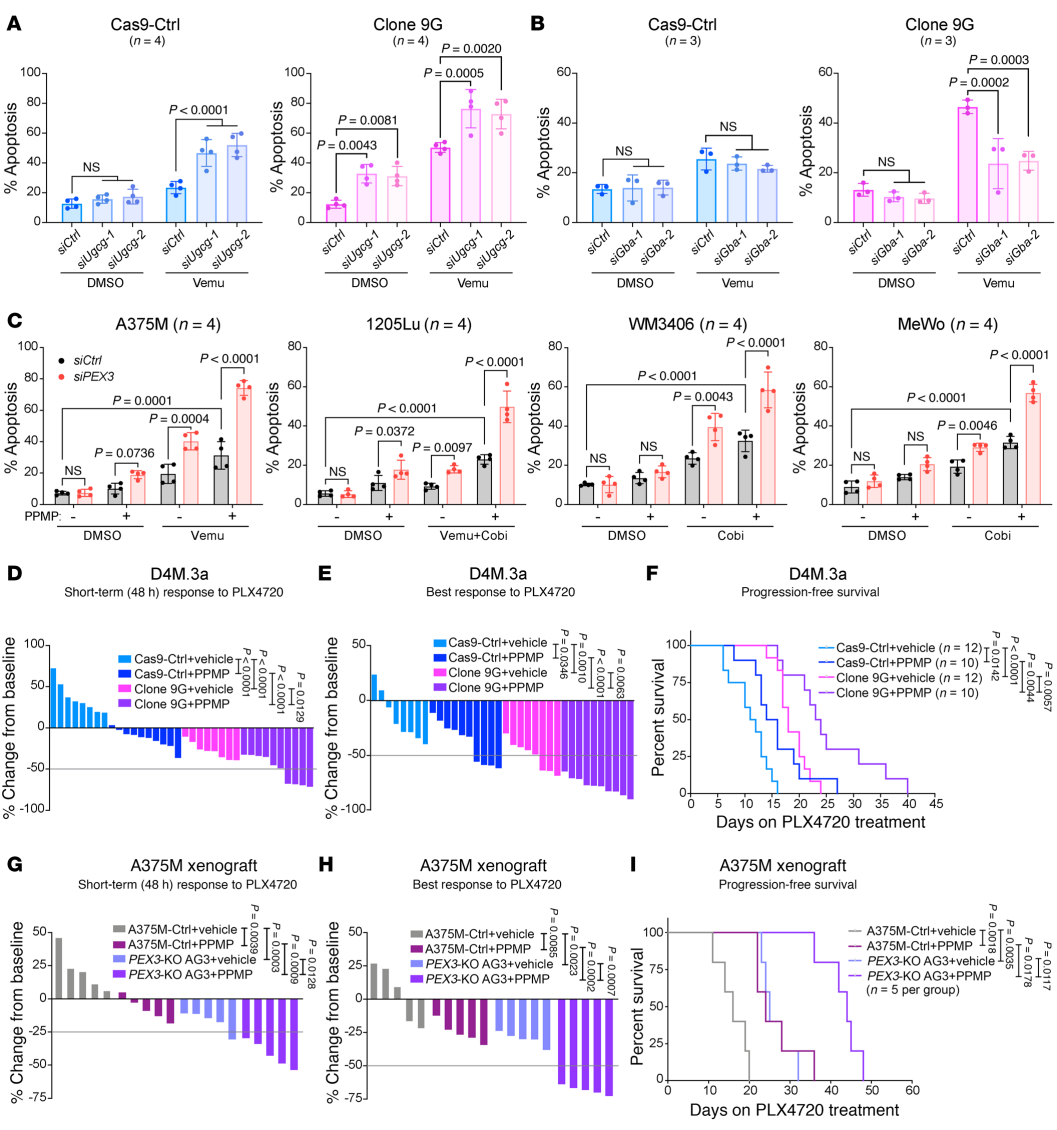
Dual blockade of PEX3 and UGCG sensitized melanoma to MAPK inhibition. (**A** and **B**) Percentage apoptosis detected in D4M.3a Cas9-Ctrl (left) or *Pex3^+/–^* clone 9G (right) cells, following (**A**) *Ugcg* or (**B**) *Gba* knockdown and treatment with vemu or DMSO control (*n* = 4 for **A**, *n* = 3 for **B**). (**C**) Percentage apoptosis detected in human melanoma cells following *PEX3* knockdown and treatment with indicated MAPK inhibitors and/or D,L-threo-PPMP (PPMP). Equal volumes of DMSO were added to the control groups (*n* = 4). Detailed treatment and timeline are presented in Supplemental Table 1. Data in **A**–**C** represent mean ± SD. (**D** and **E**) Waterfall plots showing (**D**) the STR and (**E**) the BR of D4M.3a Cas9-Ctrl– or 9G-derived melanomas to PLX4720 alone or PLX4720 combined with PPMP. (**F**) Kaplan-Meier curves showing PFS of mice bearing D4M.3a Cas9-Ctrl– or 9G-derived melanomas, treated with PLX4720 alone or PLX4720 combined with PPMP. (**G** and **H**) Waterfall plots showing (**G**) the STR and (**H**) the BR of A375M-Ctrl– or *PEX3-*KO AG3–derived melanomas to PLX4720 alone or in combination with PPMP. (**I**) Kaplan-Meier curves showing PFS of mice bearing A375M-Ctrl– or *PEX3-*KO AG3–derived melanomas, treated with PLX4720 alone or PLX4720 combined with PPMP. In **D**–**I**, the number of biological replicates (mice) is indicated in each graph. Detailed experimental timelines are shown in Supplemental Figure 4, G and J. Values in **D**, **E**, **G**, and **H** represent percentage volume change of each tumor from baseline. Significance assessed with 2-way ANOVA (**A**–**E**, **G**, and **H**) or log-rank test (**F** and **I**).

Having shown that *Pex3^+/–^* cells have a high level of ceramides and undergo apoptosis in response to further ceramide increases (Figure 2, F and G), we next sought to exploit this metabolic vulnerability for therapeutic intervention. Melanoma cells with reduced peroxisomes are characterized by increased levels of HexCers (Figure 2F and Supplemental Figure 3D), which include glucosylceramides (GluCers) and galactosylceramides (GalCers) (Supplemental Figure 3B). Given that GluCers can be prosurvival in melanoma cells (35), we posited that melanoma cells with compromised peroxisomes rely on the UGCG-catalyzed ceramide-to-GluCer metabolism as a prosurvival mechanism. Dual blockade of PEX3 and UGCG would thus be anticipated to result in a greater clinical benefit. Indeed, knockdown of *Ugcg* significantly increased apoptosis in treatment-naive *Pex3^+/–^* 9G cells (Figure 3A), indicating that these cells are reliant on UGCG-mediated ceramide clearance for survival. Additionally, knockdown of *Ugcg* further potentiated the response of the *Pex3^+/–^* 9G cells to vemu, resulting in approximately 80% cell death within 24 hours of MAPK inhibition (Figure 3A). Consistent with ceramide being proapoptotic (Figure 2G), *Ugcg*-silenced Cas9-Ctrl D4M.3a cells were also sensitized to vemu-induced apoptosis (Figure 3A). Conversely, silencing *Gba*, which encodes the enzyme catalyzing the reverse GluCer-to-ceramide metabolism (Supplemental Figure 3B), protected *Pex3^+/–^* 9G cells, but not Cas9-Ctrl cells, from vemu-induced apoptosis (Figure 3B). To verify these results in human melanoma cells, we generated *PEX3*-knockout (*PEX3*-KO) A375M cells and similar data were obtained as in our murine models (Supplemental Figure 4, A–C).

We next tested the impact of blocking UGCG enzymatic activity on MAPK-targeted therapy response using the inhibitor D,L-threo-PPMP (PPMP), which induced endogenous ceramide levels, decreased HexCers (Supplemental Figure 4D) (36, 37), and increased peroxisome abundance (Supplemental Figure 4E). *PEX3* knockdown combined with PPMP potentiated MAPKi-induced apoptosis in our melanoma cell lines (Figure 3C). Similarly, PPMP+vemu led to the highest level of cell death in the *PEX3*-KO A375M cells (Supplemental Figure 4F).

We next evaluated the impact of reducing peroxisomes in melanoma cells on their response to combined UGCG inhibition and MAPKi in vivo. Murine and human peroxisome-deficient cells showed delayed tumor initiation compared with their control counterparts (Supplemental Figure 4, G, H, J, and K). When melanomas reached a volume of 200 mm^3^, mice were administered a PLX4720 diet, and PPMP therapy was initiated (Supplemental Figure 4, G and J). While both the D4M.3a Cas9-Ctrl– and A375M-Ctrl–derived melanomas continued increasing in size 2 days following PLX4720 monotherapy, the addition of PPMP rapidly potentiated their short-term response to PLX4720 (Figure 3, D and G). The long-term treatment with combined PPMP and PLX4720 significantly improved the BR and PFS in the D4M.3a Cas9-Ctrl group, a phenotype that was much stronger in mice bearing *Pex3^+/–^* 9G–derived tumors (Figure 3, E and F). Similarly, the most improved BR and PFS were observed in the A375M *PEX3*-KO group when mice were treated with PPMP+PLX4720, compared with all other arms (Figure 3, H and I). PPMP treatment, alone or in combination with PLX4720, did not cause overt toxicity (Supplemental Figure 4, I and L). Together, our mouse modeling supports the clinical potential of blocking UGCG and PEX3 to enhance antitumor responses to MAPK-targeted therapy.

### Peroxisome and UGCG functions are required for MAPKi-tolerant CD36^+^ persister melanoma cells.

**Figure 4:**
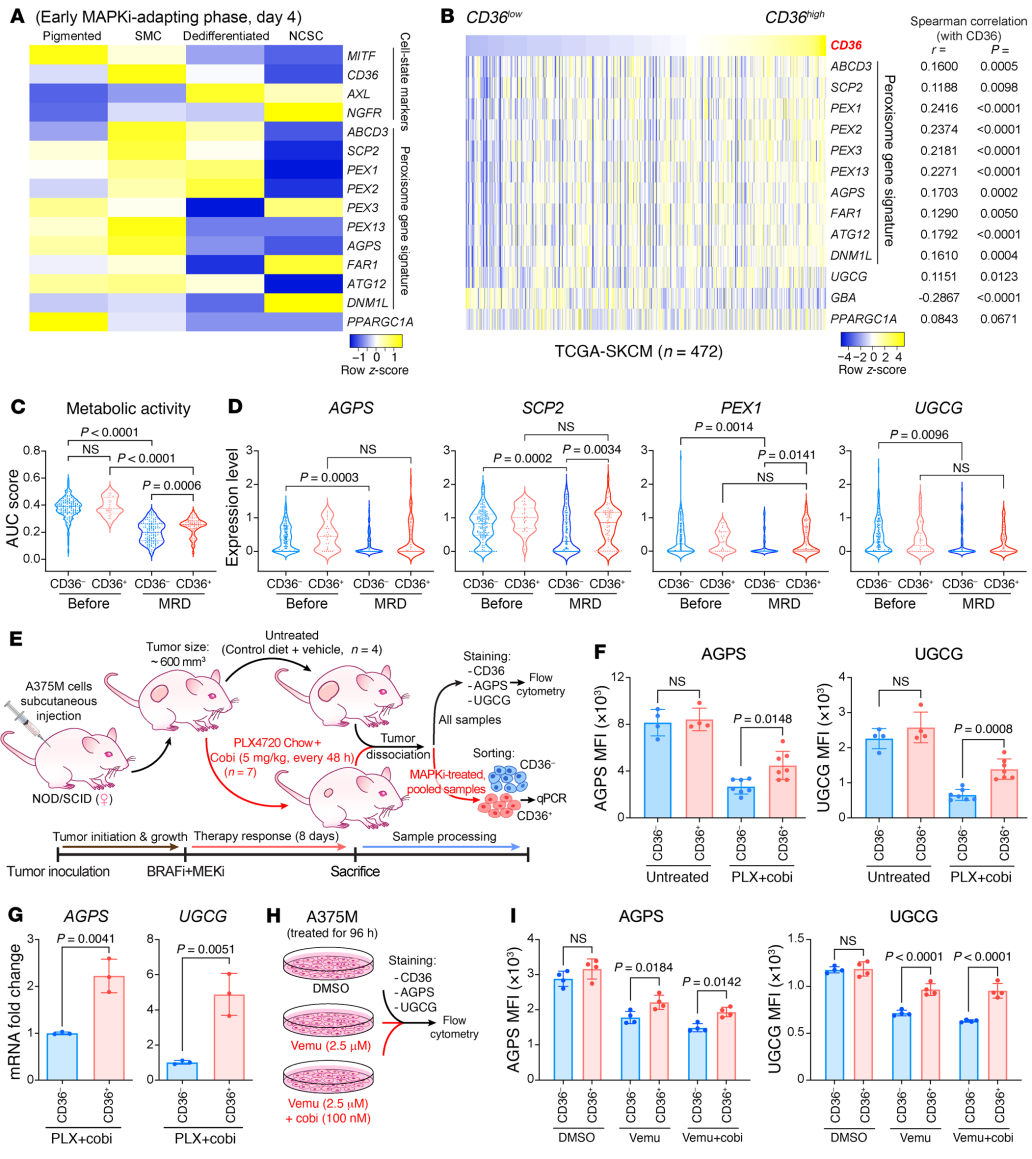
CD36^+^ MAPKi-tolerant melanoma cells have retained peroxisome levels and UGCG. (**A**) Heatmap showing relative expression of indicated melanoma cell-state-specific markers, a panel of peroxisomal genes, and *PPARGC1A* in 4 drug-tolerant melanoma populations in the MEL006 PDX model during early dabrafenib+trametinib treatment (day 4). (**B**) Expression of *CD36* and a peroxisomal gene signature, *UGCG*, *GBA*, and *PPARGC1A* (HTSeq-FPKM) in the GDC TCGA Melanoma data set (SKCM, *n* = 472). Spearman’s rank-order. (**C** and **D**) Violin plots of scRNA-seq data highlighting the distribution of (**C**) a gene signature indicating cancer cell metabolic activity and (**D**) indicated peroxisomal genes and *UGCG* in CD36^–^ (<2.2) versus CD36^+^ (≥2.2) cells, before or after dabrafenib+trametinib treatment for 28 days (MRD). (**E**) Schematic of experimental design for panels **F** and **G**. (**F**) Relative expression of AGPS (left) and UGCG (right) in CD36^–^ and CD36^+^ cell populations isolated from A375M melanoma xenografts following vehicle (*n* = 4) or PLX4720+cobi (*n* = 7) treatment for 8 days. (**G**) Fold change in *AGPS* (left) and *UGCG* (right) transcripts in CD36^+^ cells relative to CD36^–^ cells isolated from A375M melanoma xenografts following PLX4720+cobi treatment for 8 days. *RPLP0* was used as a reference gene. Cells from a total *n* = 7 tumors were pooled before sorting. Two-sided unpaired *t* test. (**H**) Schematic of experimental design for **I**. (**I**) Relative expression of AGPS (left) and UGCG (right) in CD36^–^ versus CD36^+^ A375M cells following treatment with DMSO, vemu (2.5 μM), or vemu (2.5 μM) combined with cobi (100 nM) for 96 hours. Significance assessed with 2-way ANOVA (**C**, **D**, **F**, and **I**). Data in **F**–**I** represent mean ± SD.

Cotargeting PEX3 and UGCG not only potentiated melanoma response to MAPK inhibition but also delayed the onset of resistance (i.e., prolonged PFS) (Figure 3). We posit that this strategy may eliminate specific populations of MAPKi-persister melanoma cells present in minimal residual disease (MRD), which ultimately lead to the development of resistance (19, 38). Rambow et al. identified 4 MAPKi-tolerant melanoma states in a patient-derived xenograft (PDX) model, termed pigmented, starved-like melanoma cells (SMCs), dedifferentiated (invasive), and neural crest stem cells (NCSCs) (19). Reanalysis of the Rambow scRNA-seq data set revealed that, among these drug-tolerant states present during the early MAPKi-adapting phase, a peroxisomal gene signature was specifically enriched in the SMC state that is marked by the expression of the fatty acid transporter CD36 (Figure 4A). Our analysis is consistent with prior work wherein the transcript levels of FAO, which encompassed both mitochondrial and peroxisomal enzymes, were found to characterize the SMC population (8). Importantly, in our analysis, the expression of peroxisome signature genes and *UGCG* were positively correlated with *CD36* expression in the human melanoma data set of The Cancer Genome Atlas (TCGA). In contrast, *GBA* and *CD36* expression was negatively correlated (Figure 4B). Notably, our analysis revealed that *PPARGC1A*, critical for mitochondrial metabolism–mediated MAPKi resistance in a subset of melanoma cells (5, 24), was predominantly expressed in the pigmented MITF^hi^ cell state and showed no correlation with *CD36* in TCGA data set (Figure 4, A and B). The SMC and pigmented melanoma cell states are reliant on altered metabolism for their drug tolerance (7, 24). We thus hypothesized that the CD36^+^ SMC state is distinguished from the mitochondria-dependent pigmented state by their reliance on a peroxisome/UGCG-dependent mechanism.

**Figure 5:**
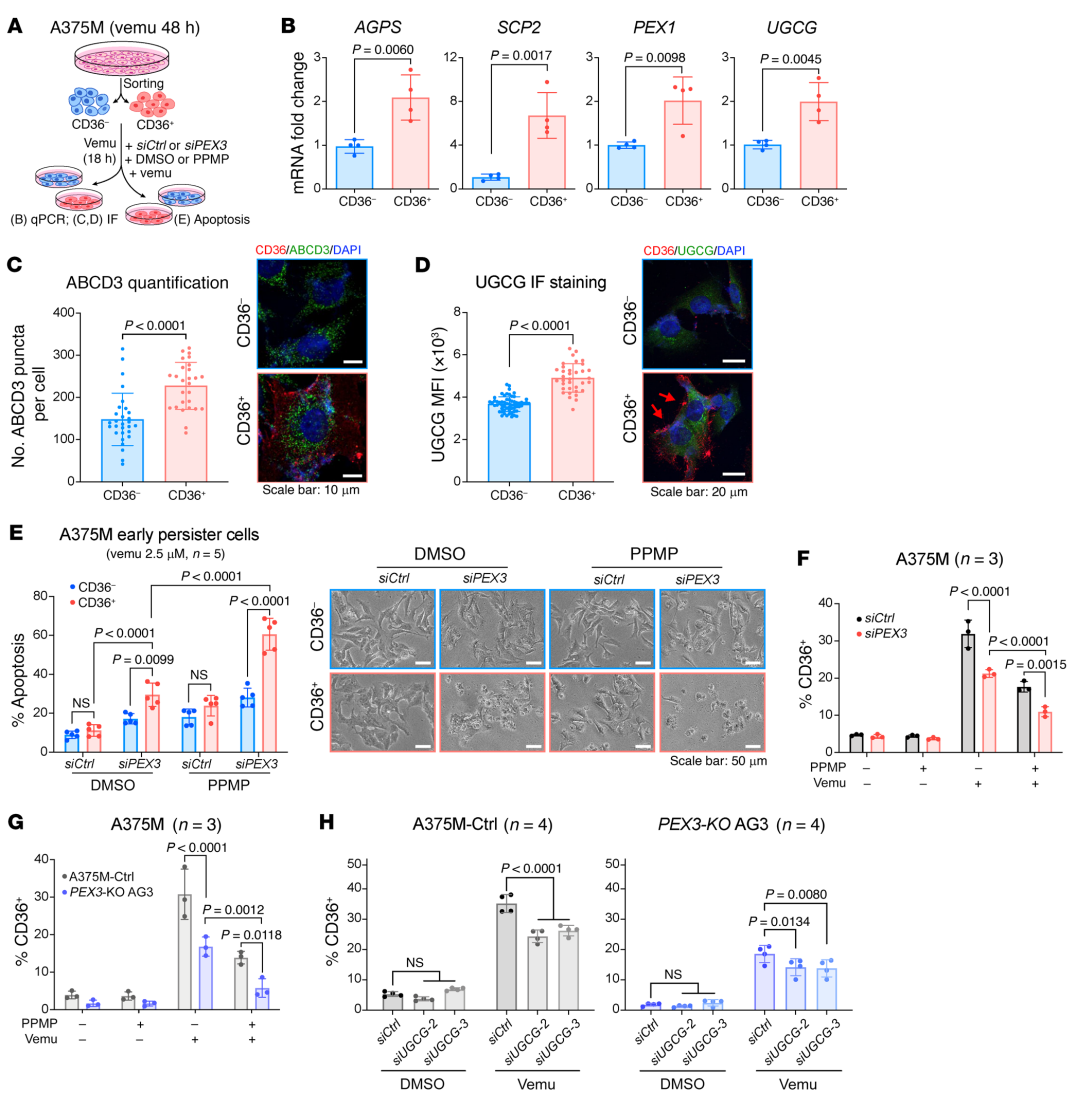
Peroxisomes and UGCG are required for survival of CD36^+^ MAPKi-tolerant melanoma cells. (**A**) Schematic of experimental design for (**B**–**E**). A375M cells were treated with vemu (2.5 μM) for 48 hours before CD36 staining and subsequent sorting. (**B**) Fold change in the indicated mRNAs in vemu-exposed CD36^+^ A375M cells relative to CD36^–^ A375M cells, normalized to *RPLP0* as a reference gene (*n* = 4). The *SCP2* primers amplify the N-terminus of the transcript initiated from the proximal promoter, encoding the peroxisome-specific protein SCPx. (**C**) Number of ABCD3 puncta and (**D**) relative UGCG expression in vemu-exposed CD36^+^ versus CD36^–^ A375M cells. Representative immunofluorescent staining for (**C**) CD36 (red), ABCD3 (green), and DAPI (blue) or (**D**) CD36 (red), UGCG (green), and DAPI (blue) are presented. Red arrows highlight cells stained positive for CD36. (**E**) Percentage apoptosis (left) and representative images (right) of vemu-exposed CD36^+^ versus CD36^–^ A375M cells following *PEX3* knockdown and/or PPMP treatment (*n* = 5). Scale bars: 10 μm (**C**), 20 μm (**D**), and 50 μm (**E**). (**F**–**H**) Percentage of CD36^+^ populations in (**F**) A375M cells following *PEX3* knockdown and the indicated treatment (*n* = 3), (**G**) A375M-Ctrl versus *PEX3-*KO AG3 cells upon indicated treatment (*n* = 3), or (**H**) A375M-Ctrl versus *PEX3-*KO AG3 cells following *UGCG* knockdown and subsequently treated with vemu (*n* = 4). Significance assessed by 2-sided unpaired *t* test (**B**–**D**) or 2-way ANOVA (**E**–**H**). Data represent mean ± SD.

We next confirmed that *CD36* marker expression was sufficient to identify the SMC population, which was originally defined by the high expression of a subset of SMC signature genes, including *CD36* (termed “SMC AUCell score”) (19). By setting a normalized *CD36* expression of 2.2 or higher as a cutoff (considered as CD36^+^) (Supplemental Figure 5A), we were able to highlight almost identical cell populations that were defined by SMC AUCell score (≥0.05) in the Rambow 2018 data set (Supplemental Figure 5B). These CD36^+^ cells and SMC populations also showed similar abundance throughout the drug response (Supplemental Figure 5C). Although dramatic decreases in the cancer cell metabolism gene signature ensue with combined BRAF/MEK inhibition, indicative of an overall low metabolic activity in MRD (19), this metabolic signature was decreased to a significantly lesser extent in the CD36^+^ cells, compared with the CD36^–^ subpopulation (Figure 4C). Consistent with these results, while peroxisome signature genes (*AGPS*, *SCP2*, *PEX1*) and *UGCG* expression were significantly decreased in the CD36^–^ cells, the expression of those genes was retained in the CD36^+^ population (Figure 4D). We further verified the robustness of these data using the A375M xenograft model. Mice bearing A375M-derived tumors were administered combined BRAFi/MEKi therapy (PLX4720 diet+cobi) for 8 days, a regimen to which melanomas fully respond (i.e., tumor shrinkage) (Figure 4E and Supplemental Figure 5D). Combined BRAFi/MEKi treatment significantly increased the CD36^+^ melanoma population (Supplemental Figure 5E). Despite an overall decrease in alkylglycerone phosphate synthase (AGPS, a peroxisomal enzyme) and UGCG following MAPK inhibition, the CD36^+^ cells had significantly higher levels of AGPS and UGCG compared with their CD36^–^ counterpart (Figure 4, F and G). Similar results were observed in vitro using vemu alone or combined with cobi (Figure 4, H and I). Together, these data suggest a potential mechanistic link between the higher metabolic activity of MAPKi-tolerant CD36^+^ SMCs and a role of the peroxisome and UGCG therein. We next formally tested whether peroxisomes and UGCG are required for MAPKi-tolerant CD36^+^ persister cells. Consistent with a previous study (7), treatment with vemu, cobi, or vemu+cobi increased the percentage of CD36^+^ A375M cells to a similar extent (Supplemental Figure 5F). We thus used vemu to model the dynamics and drug response/tolerance of these MAPKi-induced CD36^+^ cells. The abundant CD36^+^ population was observed from day 1 to day 10 after vemu treatment (Supplemental Figure 5G), recapitulating an overall trend observed in the PDX model MEL006 (Supplemental Figure 5C). After a 48-hour vemu treatment, A375M cells staining negative for both Annexin V and PI were sorted into CD36^–^ and CD36^+^ populations (Figure 5A). As expected, both populations showed lower sensitivity to vemu than the parental A375M cells (Supplemental Figure 5H). Moreover, CD36^+^ cells developed resistance within the shortest time, compared with either CD36^–^ cells or parental cells (Supplemental Figure 5H).

In agreement with our previous data (Figure 4, D–I), vemu-induced CD36^+^ A375M cells showed higher expression of peroxisomal genes (*AGPS*, *SCP2*, *PEX1*) and had more peroxisomes compared with CD36^–^ cells (Figure 5, B and C). UGCG expression was also higher in the CD36^+^ population (Figure 5, B and D). Consistently, these CD36^+^ cells exhibited increased ceramide tolerance, compared with their CD36^–^ counterparts (Supplemental Figure 5I). Supporting the essential role of peroxisomes in CD36^+^ drug-tolerant cells, the CD36^+^ population was more susceptible to apoptosis when *PEX3* was knocked down, compared with the CD36^–^ population. Moreover, this effect was potentiated when UGCG activity was simultaneously blocked using PPMP (Figure 5E). Using both genetic and pharmacological approaches, we further showed that cotargeting PEX3 and UGCG most efficiently eliminated the CD36^+^ population (Figure 5, F–H). Similar results were also observed in the NRAS-driven WM3406 cells upon MEKi treatment (Supplemental Figure 5J). Together, these data revealed a distinct peroxisome/UGCG-dependent mechanism of tolerance in the MAPKi-induced CD36^+^ melanoma persister cells. Dual blockade of PEX3 and UGCG, therefore, enhances efficacy of MAPKis by selectively eliminating these CD36^+^ persister cells.

### Increased peroxisomal activity and UGCG marks MAPKi resistance and poor outcome in melanoma.

**Figure 6:**
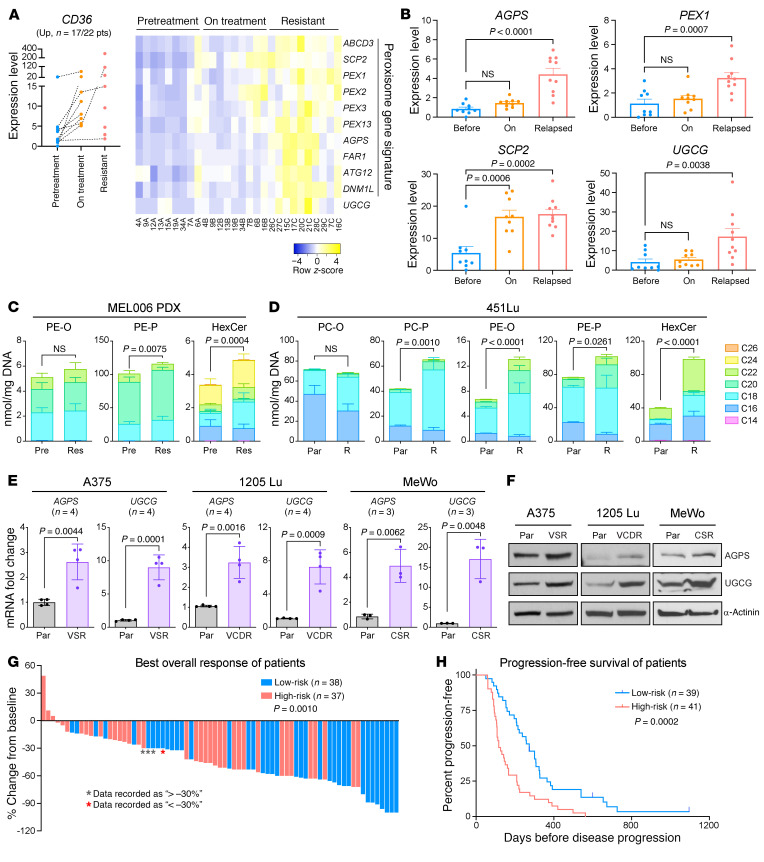
Increased peroxisomal and UGCG activity commonly occurs in melanomas that rapidly acquire resistance to MAPK-targeted therapy. (**A**) Normalized gene expression of *CD36* (left), relative expression of a peroxisomal gene signature (right), and (**B**) expression of *AGPS*, *SCP2*, *PEX1*, and *UGCG* in a cohort of melanoma samples (*n* = 17 out of a total of 22 patients, Kwong 2015 data set) collected before, while on, or relapsed on MAPK-targeted therapy showing an overall trend of *CD36* induction upon MAPKi. Data represent mean + SEM. One-way ANOVA. (**C** and **D**) Normalized abundance of indicated lipid species grouped by carbon chain length detected in (**C**) MEL006 PDX tumor samples collected before (Pre, *n* = 6) or relapsed (Res, *n* = 5) on dabrafenib+trametinib treatment, or (**D**) 451Lu parental (Par) cells versus vemu-resistant 451Lu-R cells (*n* = 3). Two-way ANOVA. PE-O, 1-alkyl,2-acylphosphatidylethanolamine; PC-O, 1-alkyl,2-acylphosphatidylcholine. (**E**) Fold change in *AGPS* and *UGCG* mRNA levels in a panel of MAPKi-resistant melanoma cells relative to their corresponding parental cells, normalized to *ACTB* as a reference gene (*n* = 4). Data in **C**–**E** represent mean ± SD. (**F**) Western blot analysis of the indicated proteins in a panel of parental versus MAPKi-resistant melanoma cells. (**G**) Waterfall plots showing the best overall response and (**H**) Kaplan-Meier curves showing PFS of melanoma patients treated with MAPK-targeted therapy. Risk score ranging between 0 and 3 was calculated based on expression of *CD36*, *AGPS*, and *UGCG* before and after treatment (see Supplemental Table 5 for detailed information). Patients were subsequently grouped into high-risk (risk score ≥2) versus low-risk (risk score ≤1). Values in **G** represent percentage response of individual patient from baseline. Significance assessed by 2-sided unpaired *t* test (**E** and **G**) or log-rank test (**H**).

Having shown that inhibiting peroxisomes and UGCG can induce death of CD36^+^ MAPKi-tolerant cells, we next interrogated the status of CD36, peroxisomes, and UGCG in melanomas from patients treated with MAPKi. We reanalyzed a publicly available RNA-seq data set (20), which included patient melanoma samples collected before, during, and after relapse of MAPKi therapy. In 77% of patients (17/22), we observed an overall induction of *CD36* expression following MAPKi treatment (Figure 6A). In this patient cohort, expression of the peroxisomal gene signature and *UGCG* was most dramatically increased in the relapsed samples (Figure 6, A and B). Notably, a small group of patients (5/22) showed decreased levels of *CD36* during treatment. No significant pattern of change in peroxisomal genes or *UGCG* was observed in this group of samples, suggesting alternative CD36^+^/peroxisome-independent mechanisms through which drug tolerance and resistance can occur (Supplemental Figure 6A). Similar trends were detected in a separate melanoma patient–derived RNA-seq data set (21, 22). Approximately 76% (19/25) of patients showed increased expression of *CD36* following MAPK-targeted therapy (Supplemental Figure 6B). Both peroxisomal genes (*AGPS*, *SCP2*, *PEX1*) and *UGCG* were significantly increased after therapy resistance (Supplemental Figure 6C). In this cohort, a slight decrease in *PEX1* and *UGCG* expression was observed in samples collected during therapy, consistent with the scRNA-seq data (Figure 4D).

Our data support the notion that peroxisomes and UGCG are essential for the survival of MAPKi-tolerant melanomas and changes in their expression can alter lipid profiles (Figures 2–5). We next interrogated lipidomic data from a human melanoma PDX (MEL006) and cell line model (451Lu) of acquired MAPKi resistance (19, 39) to understand whether they showed lipid profiles indicative of heightened peroxisome and/or UGCG activity. Several EPL species (1-alkenyl,2-acylphosphatidylethanolamine, PE-P) were increased in the MEL006 PDX model after tumors relapsed (Figure 6C), strongly indicative of increased peroxisomal/AGPS activity. Similarly, we observed significantly increased abundance of EPL species, including 1-alkenyl,2-acylphosphatidylcholine (PC-P), 1-alkyl,2-acylphosphatidylethanolamine (PE-O), and PE-P, in the vemu-resistant 451Lu-R cells, compared with the parental 451Lu cells (Figure 6D). In addition, HexCer was significantly increased in relapsed MEL006 tumors (Figure 6C) and in the 451Lu-R cells (Figure 6D). These data are consistent with our results showing that the expression of AGPS and UGCG is upregulated in a panel of MAPKi-resistant melanoma cell lines (Figure 6, E and F, and Supplemental Figure 6D).

Finally, we assessed the potential value of *CD36*, *AGPS*, and *UGCG* as biomarkers in predicting patients’ response to MAPK-targeted therapy. We collected data from 7 independent studies assessing transcriptomic changes in matched treatment-naive versus MAPKi-treated melanomas from a total of 80 patients (20–23, 40–42). Using baseline and/or treatment-induced expression of *CD36*, *AGPS*, and *UGCG* as 3 individual risk factors, patients were categorized into high- versus low-risk groups (see Methods and Supplemental Table 5). Indeed, the high-risk group of patients, marked by high or increased expression of *CD36*, *AGPS*, and *UGCG*, showed poorer clinical response to MAPK-targeted therapy and reduced PFS compared with the low-risk group (Figure 6, G and H).

To summarize, increased peroxisomal/AGPS activity and UGCG occur in a significant proportion of relapsed melanomas, which are likely driven through the CD36^+^ drug-tolerant SMC state. This subset of melanoma patients may have limited clinical response to MAPK-targeted therapy, which can potentially be improved by combined inhibition of PEX3 and UGCG.

### Overcoming MAPKi resistance by cotargeting peroxisomes and UGCG.

**Figure 7:**
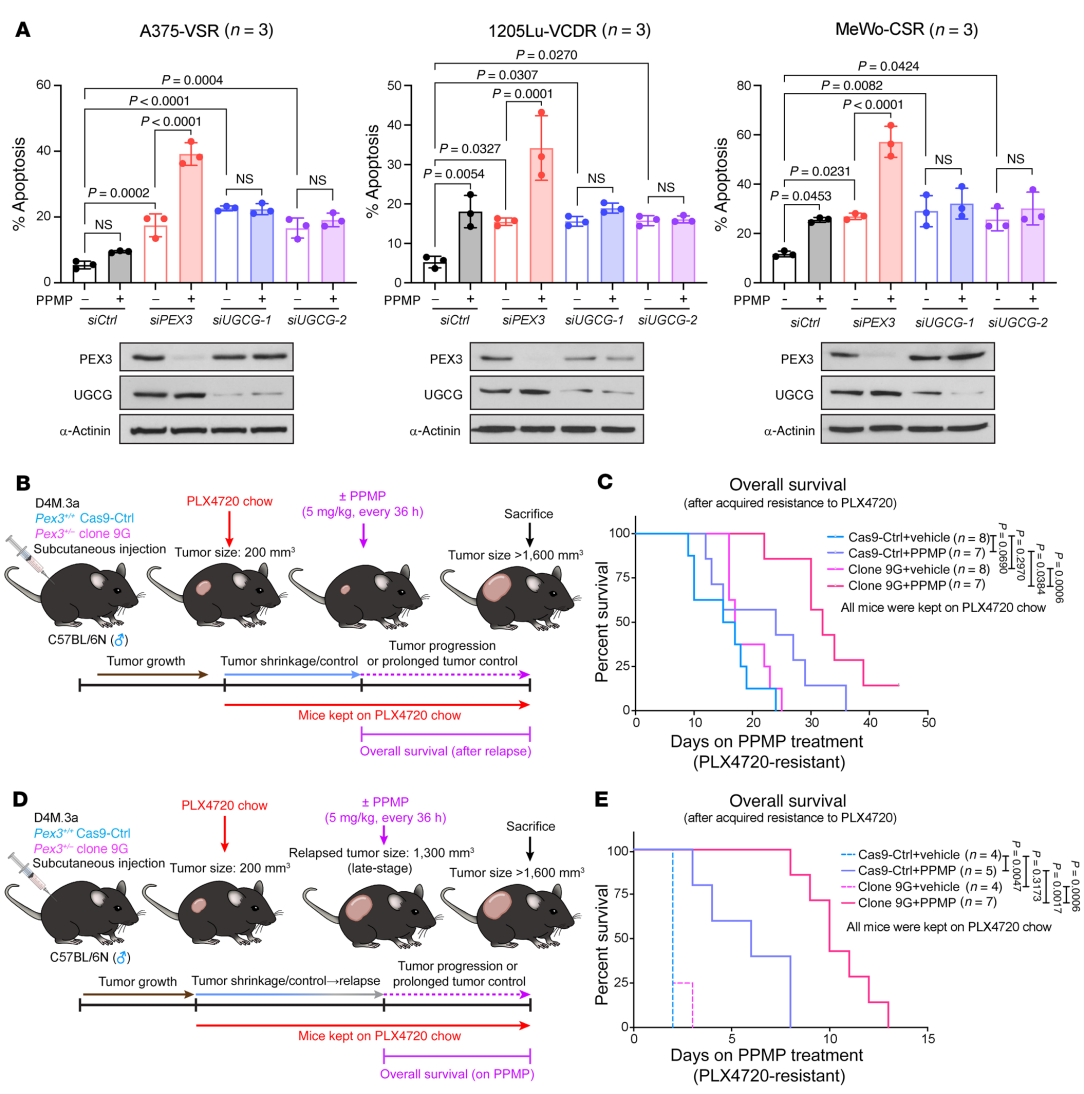
Dual inhibition of PEX3 and UGCG overcomes MAPKi resistance in melanoma. (**A**) Percentage apoptosis detected in MAPKi-resistant cells following *PEX3* or *UGCG* knockdown and treatment with PPMP. Equal volumes of DMSO were added to the control groups. Cells were maintained in the presence of indicated MAPKis (Supplemental Table 1). Detailed treatment and timeline are presented in Supplemental Table 1. Data represent mean ± SD (*n* = 3). Two-way ANOVA. (**B** and **D**) Schematic of detailed experimental design. (**C** and **E**) Kaplan-Meier curves showing overall survival (OS) of Cas9-Ctrl or *Pex3^+/–^* clone 9G tumor–bearing mice treated with PPMP or vehicle (**C**) after tumors relapsed on PLX4720, or (**E**) after relapsed tumor reached a volume of 1,300 mm^3^. All mice were kept on PLX4720 chow after PLX4720 treatment was initiated when individual tumor first reached a volume of 200 mm^3^. Number of biological replicates (mice) is indicated in each graph. Individual tumor growth curves are shown in Supplemental Figure 7, C and D. Log-rank test.

We next evaluated the efficacy of dual inhibition of PEX3 and UGCG in a panel of melanoma cell lines with acquired MAPKi resistance. These cells include previously characterized BRAF^V600E^-mutant A375, WM164, and SK-Mel-28 cells that are resistant to vemu single agent (VSR) (43, 44); newly generated BRAF^V600E^-mutant 1205Lu and SK-Mel-28 cells that are vemu+cobi dual-resistant (VCDR); and NF1-mutant MeWo cells that are resistant to cobi single agent (CSR) (see Supplemental Table 1). Knockdown of either *PEX3* or *UGCG* induced apoptosis in all drug-resistant cells that were cultured in the presence of MAPKi. The UGCG inhibitor PPMP significantly increased apoptosis in *PEX3*-silenced drug-resistant cells, without any such effect in *UGCG*-silenced cells (Figure 7A and Supplemental Figure 7A), indicating that UGCG inhibition cooperates with peroxisome reduction to overcome MAPKi resistance. Similar results were observed in *PEX19*-silenced A375-VSR cells upon PPMP treatment (Supplemental Figure 7B).

Next, we modeled the in vivo efficacy of PPMP therapy in PLX4720-resistant melanoma models with and without altered peroxisomes. Mice bearing size-matched D4M.3a Cas9-Ctrl– versus 9G-derived melanomas were kept on PLX4720 chow and PPMP treatment was initiated at the time of monitored tumor relapse (Figure 7B and Supplemental Figure 7C). While peroxisome-deficient melanomas (9G) had a delayed onset of PLX4720 resistance compared with Cas9-Ctrl–derived tumors, both groups showed similar growth rates once tumor relapse was detected (Supplemental Figure 7C, top). No significant difference in OS was observed between vehicle-treated Cas9-Ctrl versus 9G groups after acquired resistance to PLX4720 (Figure 7C and Supplemental Figure 7C). PPMP treatment led to prolonged tumor control in 43% (3/7) of mice in the PLX4720-resistant Cas9-Ctrl group (Supplemental Figure 7C). However, 100% (7/7) of mice harboring PLX4720-resistant *Pex3^+/–^* melanomas had improved tumor control and significantly increased OS on the PPMP therapy (Figure 7C and Supplemental Figure 7C). Similar results were observed in another cohort of mice where PPMP was used to treat late-stage therapy-resistant melanomas (Figure 7, D and E, and Supplemental Figure 7D). Without PPMP treatment, all mice needed to be euthanized within 48–72 hours regardless of their *Pex3* status due to their large tumor size (Figure 7E and Supplemental Figure 7D). Intriguingly, PPMP treatment improved the OS of mice bearing Cas9-Ctrl– and 9G-derived late-stage melanomas by approximately 4 and 8 days, respectively (Figure 7E and Supplemental Figure 7D). We here conclude that dual inhibition of PEX3 and UGCG has potential clinical benefit in MAPK-targeted therapy-resistant melanomas.

### The PEX3-PEX19 interaction is druggable with NNC 55-0396 and has therapeutic efficacy in melanoma.

**Figure 8:**
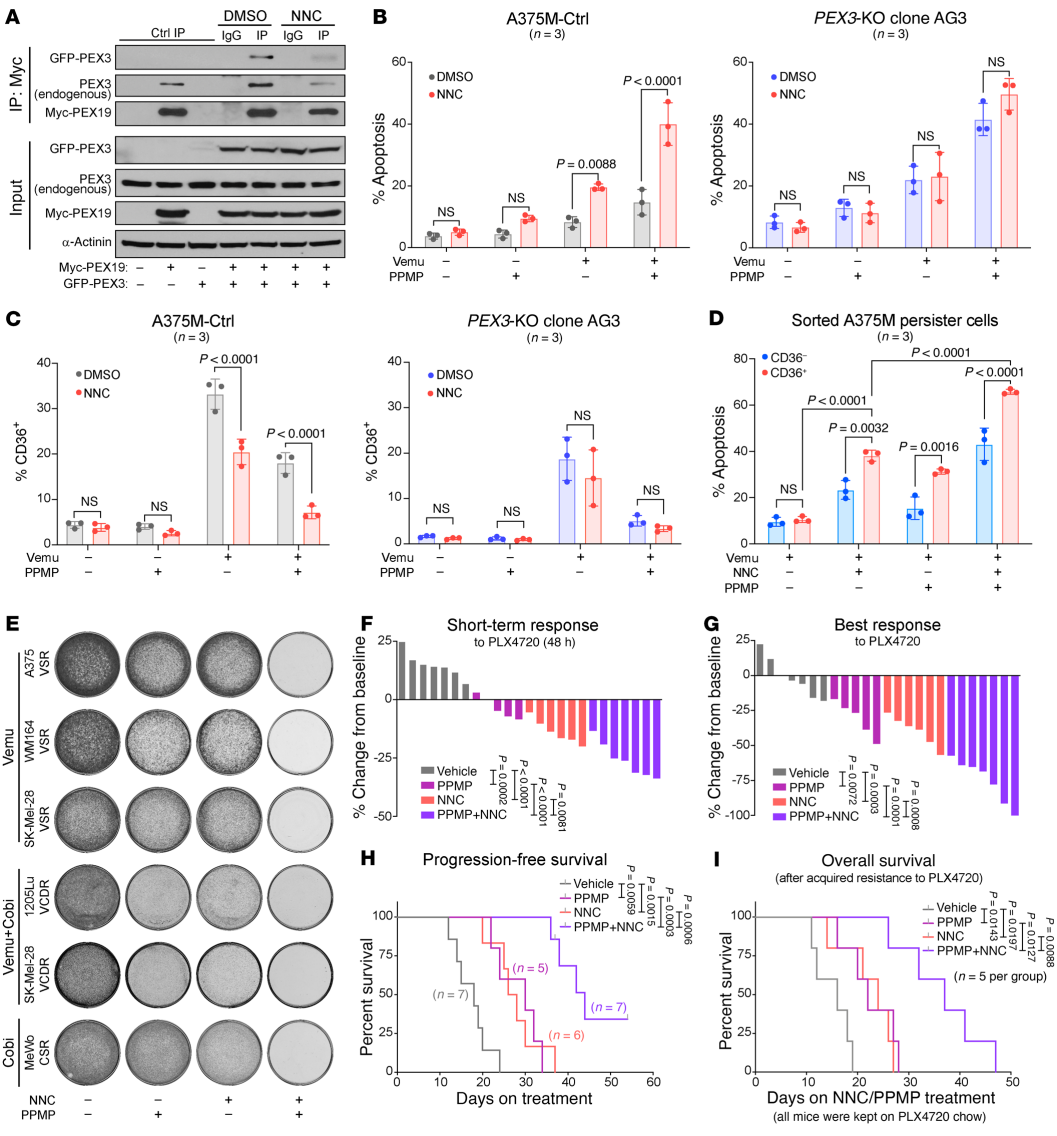
NNC 55-0396 disrupts PEX3-PEX19 interaction and cooperates with PPMP to sensitize melanoma to BRAF inhibition. (**A**) Western blot analysis of PEX3 and PEX19 from Myc co-immunoprecipitation (co-IP) of HEK-293T cells cotransfected with GFP-PEX3– and Myc-PEX19–expressing plasmids, treated with DMSO or NNC 55-0396 (NNC, 4 μM, 24 hours). Left portion displays Myc co-IP of HEK-293T cells transfected with empty vector (EV), Myc-PEX19–, or GFP-PEX3–expressing plasmid alone. Immunoblots for inputs (10% of protein from IP) are shown below (representative of *n* = 3). (**B**) Percentage apoptosis or (**C**) percentage of CD36^+^ populations detected among A375M-Ctrl cells (left) or *PEX3*-KO AG3 cells (right) following indicated treatment with NNC (4 μM), vemu, and/or PPMP. (**D**) Percentage apoptosis detected in vemu-exposed CD36^+^ versus CD36^–^ A375M cells following NNC and/or PPMP treatment (*n* = 3). Data in **B**–**D** represent mean ± SD. (**E**) Colony formation assays of a panel of MAPKi-resistant melanoma cells cultured in the presence of indicated MAPKis and treated with NNC and/or PPMP for 5 days (representative of *n* = 3). (**F** and **G**) Waterfall plots showing (**F**) the STR and (**G**) the BR of A375M-derived melanomas to PLX4720 alone or in combination with NNC, PPMP, or NNC+PPMP. Values represent percentage volume change of each tumor from baseline. (**H**) Kaplan-Meier curves showing PFS of mice bearing A375M-derived melanomas, fed with PLX4720 chow and simultaneously treated with vehicle, NNC, PPMP, or NNC+PPMP. See detailed experimental design in Supplemental Figure 9A. (**I**) Kaplan-Meier curves showing OS of A375M tumor–bearing mice treated with vehicle, NNC, PPMP, or NNC+PPMP after relapsed (PLX4720-resistant) tumor reached a volume of 400 mm^3^. Detailed experimental design and individual tumor growth curves are shown in Supplemental Figure 9, C and D, respectively. In **F**–**I**, the number of biological replicates (mice) is indicated in each graph. All mice were kept on PLX4720 chow when individual tumor first reached a volume of 200 mm^3^. Significance assessed by 2-way ANOVA (**B**–**D**, **F**, and **G**) or log-rank test (**H** and **I**).

Protein-protein interactions are potential vulnerabilities for therapeutic intervention in human disease, including cancer (45). The PEX3-PEX19 interaction is crucial for peroxisome biogenesis (46, 47), thus providing a potential intervention target with a small molecule inhibitor. NNC 55-0396 (hereafter NNC), a T-type calcium channel (TTCC) inhibitor (48), among others, was identified from a recent screen for drugs that potentially inhibit the PEX3-PEX19 interaction (Supplemental Figure 8, A and B) (49). We showed NNC decreased peroxisome numbers in A375M cells (Supplemental Figure 8C) and dramatically reduced the PEX3-PEX19 interaction (Figure 8A). Structural analysis revealed that the predicted binding site of NNC on PEX3 is in close proximity to PEX19-interacting residues (Supplemental Figure 8D) (50). Notably, overexpressing GFP-PEX3 alone resulted in degradation of PEX3, which is likely due to proteasomal degradation (Supplemental Figure 8E). However, simultaneously overexpressing PEX3 and PEX19 prevented PEX3 degradation (Figure 8A and Supplemental Figure 8E), suggesting that the PEX3-PEX19 interaction is crucial for PEX3 stability. In agreement with these data, we showed that NNC promoted PEX3 degradation within 48–72 hours (Supplemental Figure 8, E and F). Prolonged NNC treatment subsequently led to decreased expression of PEX16, PEX19, and AGPS (Supplemental Figure 8F), and increased levels of ceramides and HexCers (Supplemental Figure 8G), characteristic of cells with compromised peroxisomes (Figure 2F and Supplemental Figure 3D).

Similar to the data obtained in *PEX3*-silenced or -knockout melanoma cells, NNC treatment potentiated vemu-induced apoptosis in A375M-Ctrl cells (Figure 8B). The induction of apoptosis was significantly more robust when A375M-Ctrl cells were treated with the triple therapy of vemu and NNC in combination with PPMP (Figure 8B). Furthermore, NNC was sufficient to counteract the vemu-mediated increase in CD36-expressing cells (Figure 8C). Combined treatment with NNC and PPMP efficiently and preferentially eliminated the vemu-induced CD36^+^ population (Figure 8, C and D). Similar effects were not observed in NNC-treated A375M *PEX3*-KO (AG3) cells, supporting the idea that the observed biological effects of NNC is through a mechanism involving PEX3 (Figure 8, B and C). TTCCs, such as CaV3.1 and CaV3.2, can promote melanoma cell proliferation and protect against apoptosis (51). Thus, we tested the effect of NNC in melanoma cells expressing TTCCs or not. Knocking down CaV3.1 or CaV3.2 did not impede the ability of NNC to potentiate vemu-induced apoptosis or the NNC-mediated decrease in the CD36^+^ melanoma cell population (Supplemental Figure 8, H and I), suggesting that the effects that we observe with NNC are largely attributable to its inhibitory effect on PEX3-PEX19 binding rather than on TTCCs. In addition, NNC combined with PPMP efficiently killed a panel of drug-resistant cells cultured in the presence of MAPKi (Figure 8E), recapitulating a phenotype we observed via genetic inhibition of *PEX3* or *PEX19* (Figure 7A and Supplemental Figure 7, A and B).

**Figure 9:**
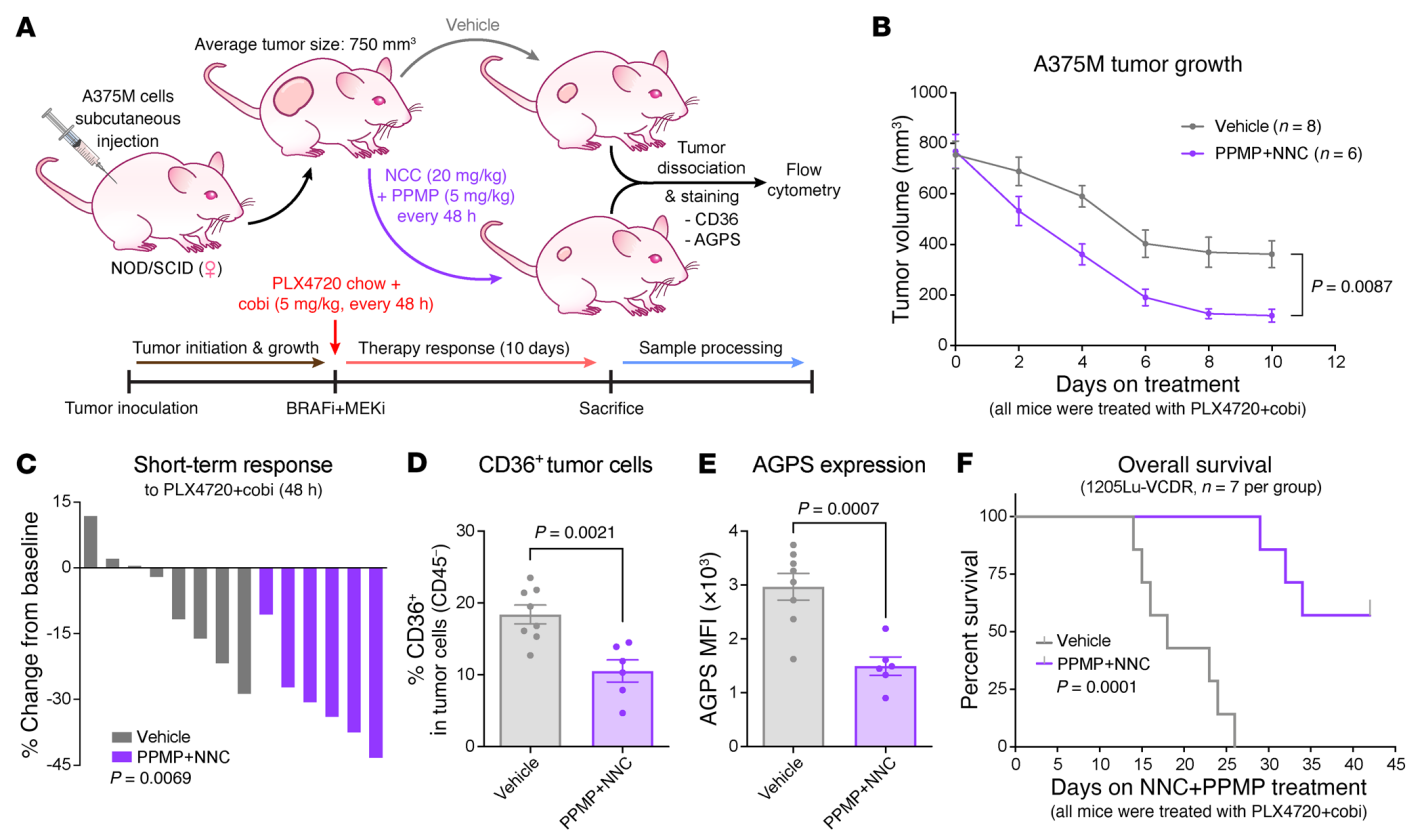
NNC 55-0396 and PPMP potentiate melanoma response to combined BRAF/MEK inhibition. (**A**) Schematic of experimental design for **B**–**E**. (**B**) Tumor growth curve and (**C**) waterfall plots showing the STR of A375M-derived melanomas to PLX4720+cobi treatment. Tumors were allowed to grow to a volume of 750 mm^3^ before indicated treatments started. (**D**) Percentage of CD36^+^ cells and (**E**) relative AGPS expression among total (CD45^–^) tumor cells, isolated from A375M-derived melanomas following indicated treatment for 10 days. (**F**) Kaplan-Meier curves showing OS of mice bearing 1205Lu-VCDR–derived melanomas treated with NNC+PPMP or vehicle. All mice were kept on PLX4720 chow and simultaneously treated with cobi. NNC+PPMP treatment started when PLX4720/cobi dual-resistant tumors first reached a volume of 250 mm^3^. Detailed experimental design and individual tumor growth curves are shown in Supplemental Figure 9, E and F, respectively. In **B**–**F**, the number of biological replicates (mice) is indicated in each graph. Data in **B**, **D**, and **E** represent mean ± SEM. Values in **C** represent percentage volume change of each tumor from baseline. Significance assessed by 2-way ANOVA (**B**), 2-sided unpaired *t* test (**C**–**E**), or log-rank test (**F**).

Next, we tested in vivo efficacy of NNC combined with PPMP and PLX4720 in the A375M xenograft model. In the first cohort, when tumors reached approximately 200 mm^3^ in size, mice were switched to the PLX4720 diet and simultaneously treated with NNC, PPMP, or NNC+PPMP (Supplemental Figure 9A). NNC+PPMP significantly potentiated the PLX4720 antitumor response in A375M-derived melanomas, compared with any other groups, a phenotype observed as early as 48 hours following the initiation of therapy (Figure 8, F and G, and Supplemental Figure 9B). Moreover, long-term administration of the triple therapy (PLX4720+NNC+PPMP) resulted in significantly improved BR and PFS, without causing any overt toxicity (Figure 8, G and H, and Supplemental Figure 9B). In a separate cohort to model therapy resistance, mice were switched to the PLX4720 diet when tumors reached approximately 200 mm^3^ in size. NNC, PPMP, or NNC+PPMP treatments were subsequently initiated when relapsed tumors reached a size of 400 mm^3^ (Supplemental Figure 9C). NNC treatment significantly prolonged tumor control and improved OS, an effect that was far more pronounced when NNC was combined with PPMP (Figure 8I and Supplemental Figure 9D). Based on these results, we next tested whether addition of a MEKi (cobi) to the triple therapy (PLX4720+NNC+PPMP) would yield similar tumor control (Figure 9A and Supplemental Figure 9E). Indeed, NNC+PPMP rapidly improved the response of A375M-derived melanomas to PLX4720+cobi treatment (Figure 9, B and C). After a 10-day course of treatment, melanomas were collected and analyzed by flow cytometry to confirm that the NNC+PPMP therapy decreased the abundance of CD36^+^ drug-tolerant cells and reduced AGPS expression (Figure 9, D and E). Finally, to test whether we have an exploitable therapy for BRAFi/MEKi dual-resistant melanomas, we injected mice with 1205Lu-VCDR cells and treated them with PLX4720+cobi when their tumors were palpable. When these drug-resistant tumors reached a size of approximately 250 mm^3^, mice were administered either NNC+PPMP therapy or vehicle control (Supplemental Figure 9E). Similar to the results obtained in the PLX4720 monotherapy-resistant cohort (Figure 8I and Supplemental Figure 9D), NNC+PPMP continuously inhibited the growth of BRAFi/MEKi dual-resistant melanomas for at least 4 weeks and dramatically improved OS (Figure 9F and Supplemental Figure 9F). Together, these data provide a proof of concept that combining NNC, a drug that disrupts the PEX3-PEX19 interaction, with a UGCG inhibitor is efficacious in sensitizing melanoma to MAPK-targeted therapy.

## Discussion

In this present study, we showed that compromising peroxisome biogenesis by targeting PEX3 (or PEX19) sensitizes melanoma to MAPK pathway inhibition and overcomes MAPKi resistance, a phenotype that is more robust when UGCG is simultaneously inhibited. Mechanistically, we identified 2 lipid pathways, the peroxisome/AGPS-driven EPL biosynthesis pathway, and the UGCG-catalyzed ceramide-to-GluCer pathway, that collaboratively regulate ceramide homeostasis and mediate drug tolerance in a subset of CD36^+^ persister cells and melanoma cells with acquired MAPKi resistance. Furthermore, we identified NNC as an inhibitor of the PEX3-PEX19 interaction, with its potent antitumor effects demonstrated in preclinical melanoma models.

Melanomas typically exhibit high intratumoral heterogeneity and cancer cell plasticity, ultimately favoring metastasis and therapeutic resistance (2, 3, 19, 38). Metabolic reprogramming represents an important mechanism by which melanoma cells switch between different states and quickly adapt to therapy-induced stress conditions (5, 24). For example, the PAX3/MITF/PGC1α axis promotes MAPKi tolerance in a subset of melanoma cells through a mitochondria-mediated metabolic shift (6, 19, 52, 53). Blockade of mitochondrial biogenesis combined with MAPKi induced apoptosis in these MITF^hi^/pigmented melanoma cells (5). These studies provide evidence that targeting cell-state-specific metabolism is a promising strategy to selectively kill drug-tolerant melanoma cell subpopulations. Importantly, our work revealed a distinct metabolic rewiring mechanism harnessed by a different subset of melanoma cells, namely the CD36^+^ SMCs, to mediate their tolerance to MAPK-targeted therapies (Figures 4 and 5). The CD36^+^ SMC state is an attractive therapeutic target in melanoma, as it represents a significant subset of drug-tolerant melanoma cells, accounting for approximately 20%–80% of the population persisting from the early MAPKi-response phase to MRD (7, 19). Both CD36^+^ SMC and pigmented melanoma cell states can rely on altered metabolism for their drug tolerance (7, 24). Previous studies suggest that SMCs rely on increased FAO, mediated by PPARα, for their survival during MAPK inhibition (7). Another study identified the peroxisomal enzyme ACOX1, a downstream target of PPARα, as a mediator of drug tolerance generally in melanoma persister cells (8). Although the latter study revealed an enrichment of FAO-related genes in SMCs, including a subset of peroxisomal enzymes, the authors did not explore the role of the PPARα/ACOX1 axis specifically in the CD36^+^ SMC population (8). Notably, the FAO-related genes that were enriched in their persister cells (total of 26 genes) also included 11 mitochondria-specific enzymes and *PPARGC1A* (encoding PGC1α) (8), a key regulator of mitochondria-dependent metabolic rewiring that functions predominately in a distinct MITF^hi^/pigmented melanoma persister cell state (Figure 4A) (6, 19, 52, 53). Using CIBERSORTx (54), we showed that the enrichment of peroxisomal genes in MAPKi-treated melanoma samples (Figure 1A) is indeed associated with SMC induction (Supplemental Figure 10A). Approximately 41% (19/46) of patients showed an increase in SMCs (>1.5-fold increase) following MAPKi treatment (Supplemental Figure 10A, left). Interestingly, an enrichment of the KEGG peroxisome gene signature was observed in approximately 95% (18/19) of patients in this group. Conversely, within patients with no change or decrease in SMC abundance after MAPK-targeted therapies, only 57% (8/14) and 46% (6/13) showed enriched KEGG peroxisome gene signature, respectively (Supplemental Figure 10A, right). Our data further demonstrated that the CD36^+^ SMCs utilize a peroxisome/UGCG-dependent mechanism for their lipid metabolic rewiring and drug tolerance, distinguishing them from the aforementioned mitochondria/PGC1α-dependent MITF^hi^/pigmented state. Dual blockade of PEX3 and UGCG effectively eliminated CD36^+^ cells, leading to a dramatic induction of apoptosis in MAPKi-treated melanoma cells and improved response to MAPKi and PFS in our preclinical models. Conversely, blocking mitochondrial OXPHOS with oligomycin potentiated melanoma cell response to vemu without affecting CD36^+^ cell abundance (Supplemental Figure 10B), further indicating that the peroxisome/UGCG-mediated lipid metabolism and the mitochondria-mediated OXPHOS independently promote drug tolerance in different melanoma cell subpopulations. Therefore, combined inhibition of both metabolic pathways can potentially work in concert to achieve better clinical responses to MAPKis (Supplemental Figure 10C). These data further support the notion that targeting cell-state-specific metabolism is an effective way to potentiate drug response. It is also worth mentioning that, while CD36 is a hallmark of the SMC state, it is not functionally associated with the increased FAO that allows SMCs to survive under MAPKi-induced metabolic stress (7). For example, knocking out *CD36* did not alter fatty acid uptake or FAO rate in MAPKi-treated A375 cells. Using a fatty acid transport protein inhibitor, Aloia et al. further showed that CD36 does not function as a fatty acid transporter in MAPKi-induced FAO (7). Therefore, existing inhibitors or neutralizing antibodies against CD36 might not be sufficient to eliminate the SMC population. Together, these data highlight the significance of combining small molecules that disrupt PEX3-PEX19 binding and UGCG inhibitors to target CD36^+^ persister melanoma cells. Finally, interrogation of TCGA data sets revealed that the expression of the peroxisome marker *ABCD3* and *UGCG* was also positively correlated with *CD36* expression in other tumor types (Supplemental Figure 10D), suggesting that combined inhibition of peroxisomes and UGCG may have broader clinical utility.

Notably, the use of dual BRAF and MEK inhibition still comes with some clinical challenges. For example, only patients with class 1 *BRAF-*mutant melanomas respond to current targeted therapy agents, and acquired resistance invariably occurs, which may also confer cross-resistance to immunotherapy through MAPKi-driven phenotype switching/dedifferentiation (2, 3). Our data showed that cotargeting PEX3 and UGCG could (a) sensitize *RAS*-mutant and *NF1*-mutant melanoma cells to MEKi-induced apoptosis, (b) delay the onset of acquired resistance in vivo, and (c) restore drug sensitivity in resistant/relapsed melanomas. We further demonstrated that dual blockade of PEX3 and UGCG eliminated MAPKi-induced CD36^+^ persister cells. Together, these data are consistent with previous observations that not only BRAFis but also MEKis induce CD36 expression in melanoma cells (7) and that the CD36^+^ SMC state is an important transitory state leading to the emergence of other MAPKi-tolerant states, which promote resistance (19). Importantly, as dedifferentiated and NCSC states are also responsible for MAPKi-induced cross-resistance to immunotherapy (2, 3), targeting these CD36^+^ cells by dual PEX3+UGCG inhibition could also potentially sensitize melanoma to combined immunotherapy and MAPKis and prevent cross-resistance.

Finally, peroxisomes have only recently been identified as a potential target for cancer management (9). There are no effective and bioavailable inhibitors of peroxisomes currently available for clinical use. Drug repurposing represents an attractive strategy for identifying new uses (i.e., inhibition of peroxisomes) for existing drugs (55). A recent drug screen aiming to discover inhibitors that disrupt trypanosomatid PEX3-PEX19 binding allowed us to identify NNC as an inhibitor of human PEX3-PEX19 interactions (49). NNC was originally developed, and is now commonly used, as a selective TTCC blocker, with an IC_50_ of 7 μM for inhibition of CaV3.1 TTCCs (48). We showed that NNC effectively disrupted PEX3-PEX19 interactions at 4 μM concentration and that the antitumoral effect of NNC is dependent on peroxisomal functions, but not on TTCCs. Moreover, NNC is bioavailable and demonstrates potent antimelanoma activity in combination with PPMP and PLX4720 (or PLX4720+cobi) in vivo. These data support the use of NNC as a PEX3-PEX19 binding inhibitor and provide a proof of concept that pharmacological blockade of peroxisomes and UGCG represents a promising strategy in melanoma.

## Methods

Additional details can be found in the Supplemental Methods.

### Mice.

Male C57BL/6N mice (6–8 weeks old) were purchased from Charles River Laboratories. Male and female nonobese diabetic (NOD)/severe combined immunodeficiency (SCID) mice (6–10 weeks old) were gifted by Moulay Alaoui-Jamali (Lady Davis Institute, McGill University). All mice were randomized before injection. All melanoma cell lines were freshly prepared in PBS and subcutaneously injected into the right flank of mice. Tumor initiation was determined once palpable tumors were formed. Tumors were then measured in length (*L*) and width (*W*). Tumor volumes (*V*) were calculated based on the formula *V* = 3.1416/6 × *L* × *W*^2^. The STR rate was calculated based on the initial tumor volume (*V*_0_) and the tumor volume measured 48 hours after treatment (*V*_48_), using the formula STR (%) = (*V*_48_ – *V*_0_)/*V*_0_ × 100. The BR rate was calculated based on the initial tumor volume (*V*_0_) and the smallest 3 consecutive tumor volumes (*V*_min1_, *V*_min2_, *V*_min3_) measured during the course of treatment, using the formula BR (%) = ([*V*_min1_ + *V*_min2_ + *V*_min3_]/3 – *V*_0_)/*V*_0_ × 100.

Detailed descriptions of experiments involving subcutaneous injection of D4M.3a, A375M, and 1205Lu-VCDR cells, drug preparation, and treatment timeline can be found in Supplemental Methods.

### Cells and reagents.

Sources, culture conditions, and treatment timelines of human melanoma cell lines are listed in Supplemental Table 1. The SK-Mel-28-VSR and WM164-VSR cells were previously generated and described as SK-Mel-28R and WM164R, respectively (44). The vemu/cobi dual-resistant 1205Lu-VCDR cells were generated by culturing of the parental 1205Lu cells in elevated doses of vemu+cobi simultaneously. SK-Mel-28-VCDR cells were generated by exposing the vemu-resistant SK-Mel-28-VSR cells sequentially to elevated doses of cobi (with the presence of vemu). The cobi-resistant MeWo-CSR cells were generated by culturing of the parental MeWo cells in elevated doses of cobi. The murine melanoma D4M.3a cell line was a gift from C.E. Brinckerhoff (Geisel School of Medicine at Dartmouth, Hanover, New Hampshire, USA), and was cultured in Advanced DMEM/F12 media supplemented with 5% FBS, 1× GlutaMax, and 1× Pen/Strep.

### Generation of CRISPR cell lines.

CRISPR/Cas9-mediated knockout of *PEX3*/*Pex3* in A375M and D4M.3a cells was accomplished using commercially available plasmids. Predesigned sgRNAs targeting *PEX3* or *Pex3* were constructed into the pSpCas9 BB-2A-GFP (PX458) vector by the manufacturer (GenScript). A375M and D4M.3a cells were transfected with either control pSpCas9 BB-2A-GFP plasmid (Cas9-Ctrl) or *PEX3*/*Pex3*-targeting sgRNA/pSpCas9 BB-2A-GFP plasmids. Individual GFP-positive clones were sorted into single cells in 96-well plates 48 hours after transfection. Single-cell clones were expanded and subsequently validated for PEX3 status by Western blotting and sequencing.

### RNA interference.

siRNAs were transfected into cells using Lipofectamine RNAiMAX Transfection Reagent (Invitrogen, 13778) following the manufacturer’s instructions, and 18 hours later, the media were changed. All cells were harvested between 48 and 96 hours after siRNA transfection. All siRNA sequences are listed in Supplemental Table 2.

### Immunofluorescence.

One coverslip/well was placed in a 24-well plate before cells were seeded. At the time of harvesting, media were aspirated and cells were washed with PBS. Cells were fixed in 4% formaldehyde/PBS for 20 minutes at room temperature (RT) and washed with PBS. Cell membranes were permeabilized using 0.2% Triton X-100/PBS for 10 minutes. Cells were washed and blocked with 10% BSA/PBS for 1 hour at RT, and then incubated with indicated primary antibodies diluted in 2% BSA/PBS overnight at 4°C in a humid chamber. Detailed antibody information is listed in Supplemental Table 3. The next day, cells were washed and incubated with secondary antibodies for 1 hour at RT in a humid, dark chamber. Cells were then washed with PBS, followed by incubation with 1:1000 DAPI/PBS nuclear stain for 15 minutes. Cells were washed and mounted to a glass slide using ProLong gold mounting media (Invitrogen, P36930). Slides were stored in the dark until confocal fluorescence images were taken. Stacking and coloring of images were performed using FIJI software (https://github.com/fiji/fiji). ABCD3 puncta were manually counted on FIJI software using stacked images.

### Flow cytometry–based assays.

Following indicated treatments, cells were trypsinized, centrifuged at 240*g* for 5 minutes, and washed in PBS. For apoptosis detection of nonfixed cells, Alexa Fluor 647–Annexin V (Invitrogen, A23204) and Propidium Iodide (PI) Staining Solution (BD Biosciences, 556463) were used following the manufacturer’s instructions. For dichlorodihydrofluorescein diacetate (DCFDA) staining, cells were stained with 5 μM H2-DCFDA (Thermo Fisher Scientific, D399) for 30 minutes at 37°C, followed by Annexin V/PI staining. For CD36 staining, cells were stained with Brilliant Violet 421 anti–human CD36 (clone 5-271, BioLegend, 336229) antibody diluted at 1:400 in PBS containing 2% FBS for 30 minutes on ice. Cells were then washed and sorted into CD36^+^ and CD36^–^ populations, or fixed for subsequent intracellular staining. For tumor samples, freshly resected tumors were minced and digested in 4 mg/mL collagenase A (Sigma-Aldrich) at 37°C for 1 hour to obtain single-cell suspensions and were stained with indicated antibodies as well as a live/dead discrimination dye (see Supplemental Figure 11 and Supplemental Table 3). For all other panels, representative images showing gating strategy and antibody information can be found in Supplemental Figure 11. All flow cytometry experiments were conducted on an LSRFortessa (BD Biosciences).

### Western blotting and quantitative real-time PCR.

Immunoblots and qPCR were performed as previously described (44, 56). Detailed descriptions can be found in Supplemental Methods. Antibody information and primers used for qPCR are listed in Supplemental Tables 3 and 4, respectively.

### Lipidomic analyses.

From 3 separate passages per cell line, D4M.3a Cas9-Ctrl, *Pex3^+/–^* clone 6D, and *Pex3^+/–^* clone 9G cells were cultured to 70%–80% confluence, trypsinized, washed twice in PBS, pelleted in a 15 mL conical tube, and flash frozen in liquid nitrogen. The samples were homogenized and mixed with 0.9 mL MeOH/HCl (1N) (8:1), 0.8 mL CHCl_3_, and 200 μg/mL 2,6-di-*tert*-butyl-4-methylphenol (Sigma-Aldrich, B1378). The organic fractions were evaporated, reconstituted in MeOH/CHCl_3_/NH_4_OH (90:10:1.25), and lipid standards were added (Avanti Polar Lipids). Phospholipids were analyzed by electrospray ionization tandem mass spectrometry (ESI-MS/MS) on a hybrid quadrupole linear ion trap mass spectrometer (4000 QTRAP system, AB SCIEX) equipped with a TriVersa NanoMate robotic nanosource (Advion Biosciences), as described previously (39). Concentrations of lipid species were normalized to DNA quantity, and relative lipid concentrations are presented as nmol lipid per mg DNA. To obtain statistical information corresponding to fold-changes in lipids between cell lines, data were uploaded onto Perseus (MaxQuant). Values and names of lipid species were numerically and textually characterized. Numerical values were then log_2_ transformed to obtain a normal numerical distribution pattern within sample types. Zeroes were then imputed using standard settings to fall within the normal curve distribution. Triplicates were grouped according to each cell line, and a multiple-sample 1-way ANOVA statistical test was performed to determine whether lipid values differed between cell lines. A post hoc test was then conducted to determine which cell lines exhibited statistically significant (*P* ≤ 0.05) differences in log_2_-transformed lipid abundances. To determine enrichment of species between cell lines, a fold-change cutoff of 1.5 or greater and *P* value of 0.05 or less was set.

### Coimmunoprecipitation.

HEK-293T cells were transfected with plasmids overexpressing TurboGFP-tagged PEX3 (OriGene Technologies, RG202031) and Myc-DDK-tagged PEX19 (OriGene Technologies, RC201756) using Lipofectamine 2000 (Invitrogen, 11668-019). The next day, media were changed, and cells were treated with 4 μM NNC 55-0396 (TOCRIS Bioscience, 2268) or DMSO control for 24 hours. Cells were then scraped and lysed with cell lysis buffer (25 mM HEPES pH 7.5, 115 mM potassium acetate, 1 mM EDTA, 1% NP-40) supplemented with protease and phosphatase inhibitors (Roche). Equal amounts of cell lysates were incubated with anti-Myc (Cell Signaling Technology, 2276S) antibody for 1.5 hours and immunoprecipitated with 25 μL of Protein G Dynabeads (Thermo Fisher Scientific, 10004D) for 1 hour at 4°C. After washing with cell lysis buffer, the immunocomplexes were analyzed by Western blotting using anti-PEX3, anti-PEX19, and anti–α-actinin antibodies (see Supplemental Table 3).

### Access and reanalysis of previously published data sets.

Normalized scRNA-seq data from the MEL006 PDX model were downloaded from the NCBI Gene Expression Omnibus (GEO) public database, accession number GSE116237 (19). A total of 674 cells were projected onto a 2-dimensional space using t-distributed stochastic neighbor embedding (t-SNE). To determine the 4 established drug-tolerant states and cell metabolic activity, we used the AUCell algorithm based on their characteristic gene signatures, as previously described (19). *CD36* expression was further examined and cells with *CD36* expression of 2.2 or higher were considered as CD36^+^ and were highlighted in the t-SNE map.

Normalized RNA-seq data from patient samples (Kwong 2015 data set) were provided by Genevieve M. Boland (Massachusetts General Hospital, Boston, Massachusetts, USA) and can be found in the European Genome-phenome Archive (EGA S00001000992) (20). Separate RNA-seq data sets (Hugo 2015 and Song 2017) were downloaded from the GEO public database, accession numbers GSE65186 and GSE75313 (21, 22). A final RNA-seq data set from a total of 6 patients (Tirosh 2016) was directly accessed from the original publication (NIHMS791837-supplement-9 in ref. 23). Normalized and background-corrected microarray data (Kakavand 2017, Long 2014, and Rizos 2014) were downloaded from the GEO public database, accession numbers GSE99898, GSE61992, and GSE50509 (40–42).

For GSEA, RNA-seq data from matched pre- and posttreatment samples were analyzed through https://www.gsea-msigdb.org/gsea/index.jsp, using predefined gene sets (see Supplemental Table 6). For patient response, PFS, and risk analysis, only patients with gene expression (*CD36*, *AGPS*, *UGCG*) data from matched tumor samples that were collected before treatment, on treatment, and/or after relapse were further analyzed and categorized into high- versus low-risk groups using 3 risk factors: RF1, RF2 (a or b), and RF3 (a or b). Briefly, baseline expression of *AGPS* (RF2a) and *UGCG* (RF3a) before treatment and fold change in *CD36* (RF1), *AGPS* (RF2b), and *UGCG* (RF3b) expression after treatment (compared with before) were normalized into *z* scores within each data set. A high *z* score for each risk factor equals a risk score of 1. High- and low-risk groups are defined by a risk score of 2 or lower and 1 or lower, respectively. Detailed risk scores and patient information are listed in Supplemental Table 5.

### Statistics.

In vitro data are presented as mean ± SD. In vivo data are presented as mean ± SEM. Prism software (GraphPad) was used to determine statistical significance of differences. Figure legends specify the statistical analysis used. *P* values are indicated in the figures and a *P* value of less than 0.05 was considered significant.

### Study approval.

Animal experiments were conducted according to the regulations established by the Canadian Council of Animal Care, and protocols approved by McGill University Animal Care and Use Committee (no. 2015-7672).

### Data availability.

Data are available in the Supporting Data Values XLS file. Supporting analytic code for reanalysis of scRNA-seq data can be accessed at https://github.com/MMdR-lab/Huang-peroxisome-melanoma

## Author contributions

FH and SVDR designed research studies. FH, FC, MSD, KG, AT, JD, FET, JYP, ML, JHL, and PG conducted experiments. FH, FC, MSD, KG, AT, JD, FET, JYP, CG, NG, JS, and PG acquired data. FH, FC, MSD, KG, AT, JD, FET, JYP, ML, CG, NG, JS, JHL, and PG analyzed and interpreted data. FH, MSD, AT, JJM, WHM and SVDR wrote, reviewed, and/or revised the manuscript. AT, JD, and JVS provided the processed quantitative lipidomic data from the MEL006 PDX model and lipidomic data from 451Lu and 451Lu-R cells. JSJ and JVS provided administrative, technical, or material support. WHM and SVDR supervised the study.

## Supplementary Material

Supplemental data

Supplemental table 5

Supplemental table 6

Supplemental table 7

Supporting data values

## Version 1 08/24/2023

In-Press Preview

## Version 2 10/16/2023

Electronic publication
