# Supplementary material for: Peroxisome disruption alters lipid metabolism and potentiates antitumor response with MAPK-targeted therapy in melanoma
Source: J Clin Invest. 2023 Oct 16;133(20):e166644. doi: 10.1172/JCI166644 (PMC10575734; doi:10.1172/JCI166644)
Supplement: Supplemental data [file jci-133-166644-s126.pdf]

Supplemental Figures

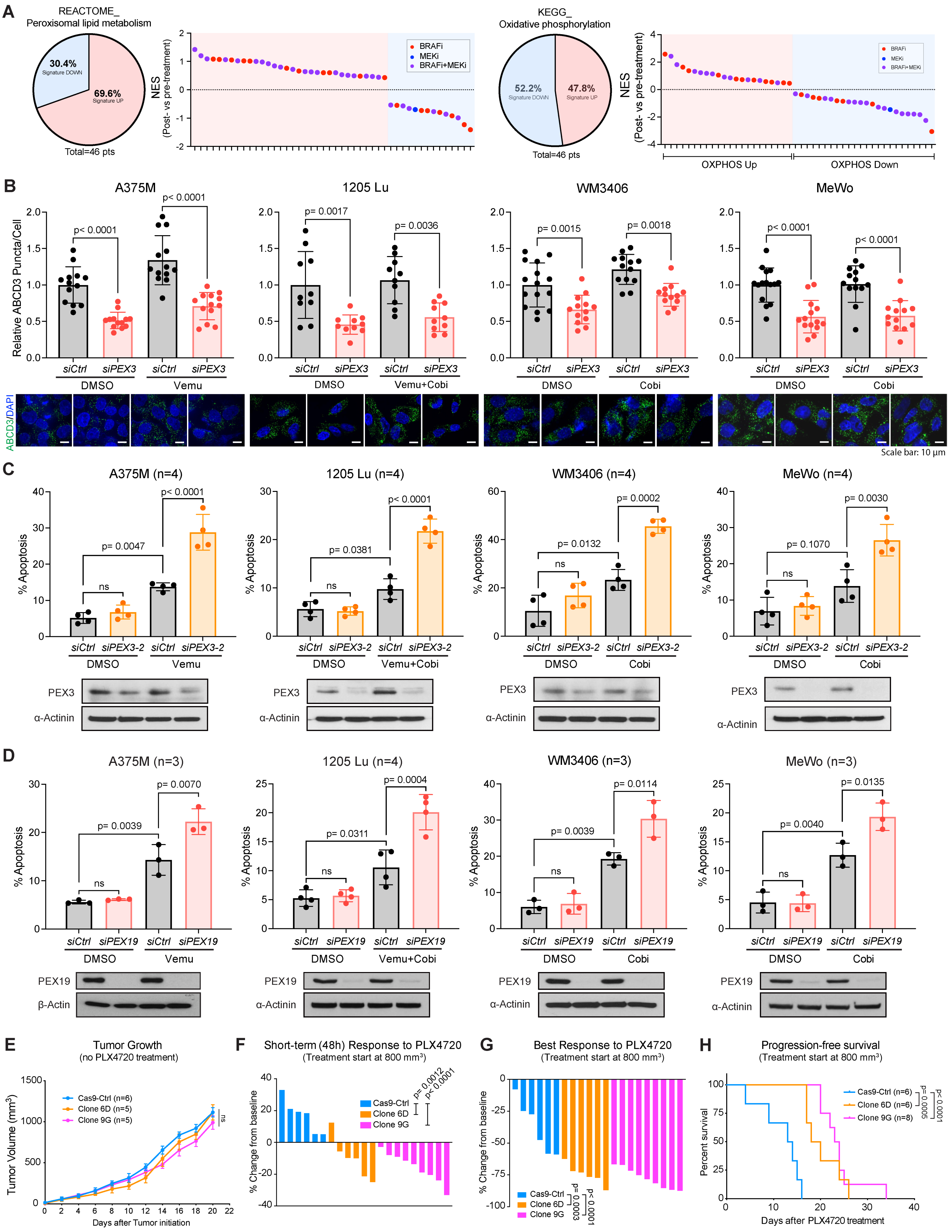

Figure S1. Compromising peroxisome biogenesis sensitizes melanoma to MAPKi-induced apoptosis.
(A) Pie charts summarizing percentage of patients (n=46) with increased or decreased expressions of indicated gene sets after treatment with MAPK-targeted therapies. Normalized enrichment scores (NES) of samples from each patient (post- versus pre-treatment) are shown. **(B)** Relative number of peroxisomes indicated by ABCD3 puncta in human melanoma cells following *PEX3* knockdown (or *siCtrl* transfection) and treatment of indicated MAPK-targeted therapy agents. Representative immunofluorescence (IF) staining for ABCD3 (green) and DAPI nuclear stain (blue) are presented (n=3). **(C, D)** Percent apoptosis measured as the sum of PI^+/Annexin V^+ and PI^-/Annexin V^+ populations (top) and western blot analysis to confirm knockdown (bottom) in human melanoma cells following **(C)** *PEX3* (*siPEX3-2*, see Table S2) or **(D)** *PEX19* knockdown (or *siCtrl* transfection) and treatment of indicated MAPK-targeted therapy agents. **(B-D)** Equal volume of DMSO was added in the control groups. Detailed treatment and timeline are presented in Table S1. Two-way ANOVA. **(E)** Tumor growth curve comparing D4M.3a Cas9-Ctrl-, 6D- and 9G-derived melanomas grown on C57BL/6N host mice fed with AIN-76A control diet. Two-way ANOVA. **(F, G)** Waterfall plots showing **(F)** the short-term response (STR, 48h after treatment initiation) and **(G)** the best response (BR) of D4M.3a Cas9-Ctrl-, 6D- and 9G-derived melanomas to PLX4720. Values represent % change from baseline. One-way ANOVA. **(H)** Kaplan-Meier curves showing progression-free survival (PFS) of mice bearing D4M.3a Cas9-Ctrl-, 6D- and 9G-derived melanomas, fed with PLX4720 chow. Log-rank test. **(F-H)** Tumors were allowed to grow to a volume of 800 mm^3 before PLX4720 treatment started. Data are presented as mean ± SD for **(B-D)** and mean ± SEM for **(E)**. Number of biological replicates is indicated in each graph.

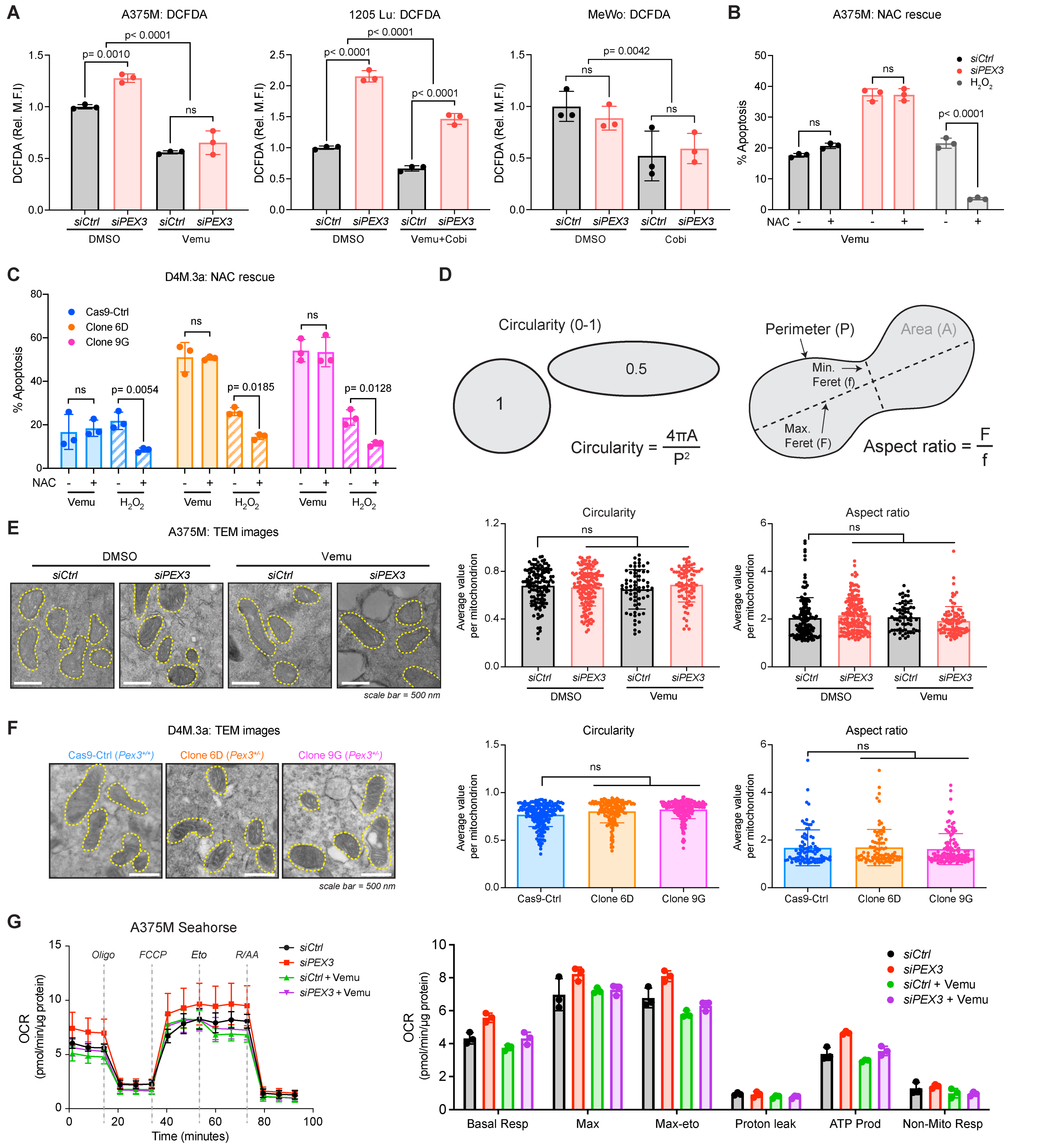

Figure S2. ROS metabolism and mitochondrial dysfunction do not explain the increased sensitivity of *PEX3*-defficient melanoma cells to MAPK inhibition.
(A) DCFDA relative mean fluorescence intensity (MFI) in human melanoma cells following *PEX3* knockdown and treatment of indicated MAPK-targeted therapy agents (n=3). Equal volume of DMSO was added in the control groups. **(B, C)** Percent apoptosis (PI+/Annexin V+, PI-/Annexin V+) detected in **(B)** *siPEX3*- or *siCtrl*-transfected A375M cells and **(C)** D4M.3a Cas9-Ctrl, 6D and 9G cells, following vemu or DMSO treatment with or without the presence of NAC. Corresponding H_2O_2-treated cells were cultured with or without NAC as positive controls (n=3). **(A-C)** Two-way ANOVA. **(D)** Schematic showing circularity (left) and aspect ratio (right) of a mitochondrion. **(E, F)** Representative electron microscope images of **(E**, left**)** *siPEX3*- or *siCtrl*-transfected A375M cells with or without vemu treatment, or **(F**, left**)** D4M.3a Cas9-Ctrl, 6D and 9G cells. The analyses of mitochondrial circularity and aspect ratio in each condition are presented (right). **(E)** Two-way ANOVA. **(F)** One-way ANOVA. **(G)** Seahorse analysis assessing mitochondrial oxygen consumption rate (OCR) in A375M cells following *siPEX3* or *siCtrl* transfection and subsequent vemu or DMSO control treatment (n=3). All data are presented as mean ± SD.

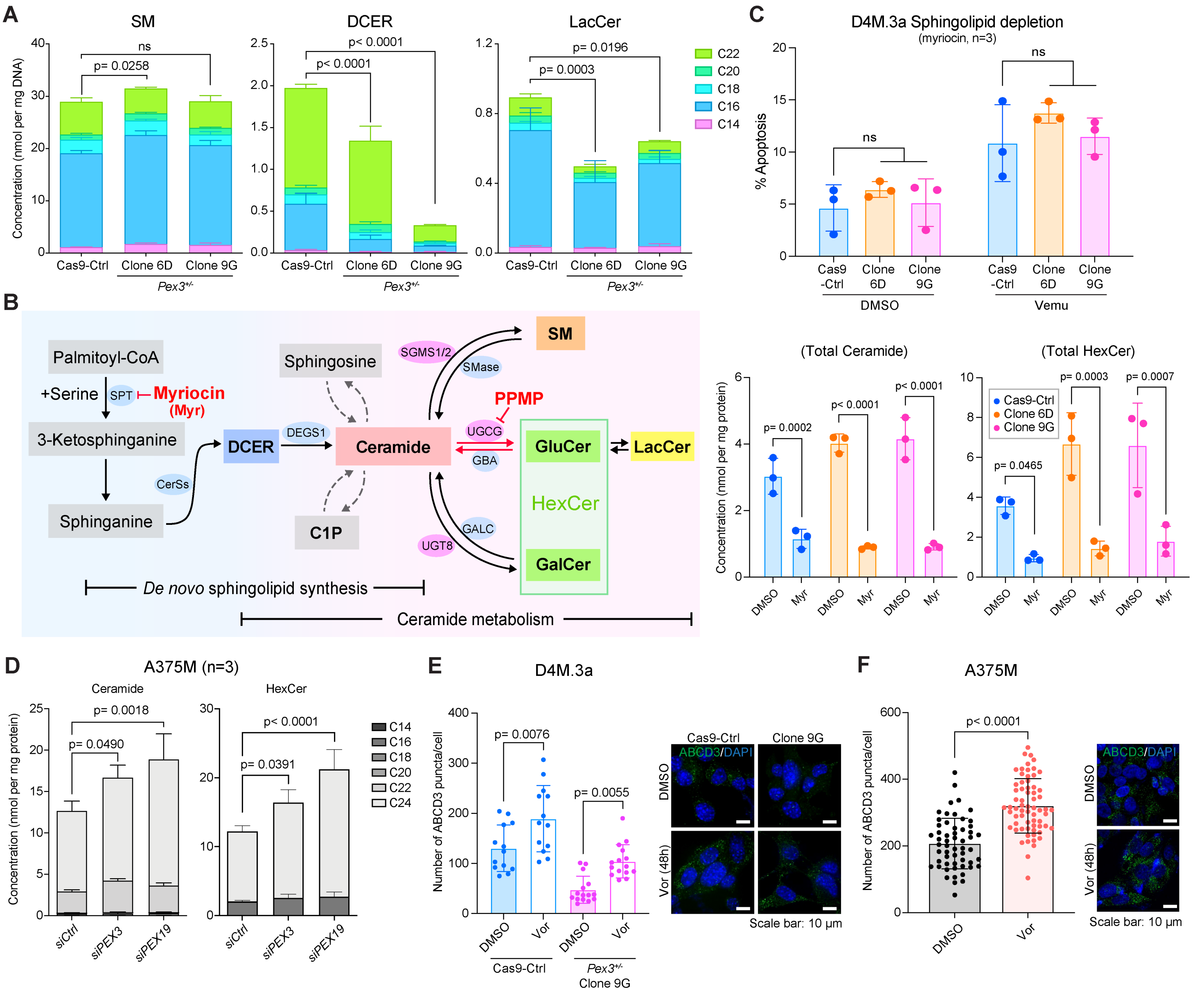

Figure S3. Disrupting peroxisome biogenesis alters sphingolipid composition in melanoma cells.
(A) Concentrations of sphingomyelins (SM), dihydroceramides (DCER), and lactosylceramides (LacCer) detected in D4M.3a Cas9-Ctrl, 6D and 9G cells (n=3). **(B)** Schematic of the *de novo* sphingolipid synthesis pathway (left) and pathways centering on ceramide metabolism (right). D,L-threo-PPMP (PPMP) blocks UGCG mediated ceramide-to-GluCer metabolism and thereby increases ceramide abundance. **(C)** Top: Percent apoptosis (PI$^+$/Annexin V$^+$, PI$^-$/Annexin V$^+$) detected in D4M.3a Cas9-Ctrl, 6D and 9G cells, pretreated with myriocin (Myr, 1µM) for 72 hours and subsequently treated with vemurafenib (vemu) or DMSO for 24 hours. Bottom: Concentrations of ceramides and HexCer in D4M.3a Cas9-Ctrl, 6D and 9G cells pretreated with DMSO or Myr for 72 hours (n=3). **(D)** Concentrations of ceramides and HexCer in A375M cells following siRNA-mediated *PEX3* or *PEX19* knockdown for 10 days (n=3). **(E, F)** Number of ABCD3 puncta in **(E)** D4M.3a (Cas9-Ctrl versus *Pex3*$^{+/-}$ Clone 9G) cells, or **(F)** A375M cells, treated with DMSO control or vorinostat (Vor, 1µM) for 48 hours. Representative IF staining for ABCD3 (green) and DAPI nuclear stain (blue) are presented. **(A, C-E)** Two-way ANOVA. **(F)** Two-sided unpaired t-test. All data are presented as mean ± SD.

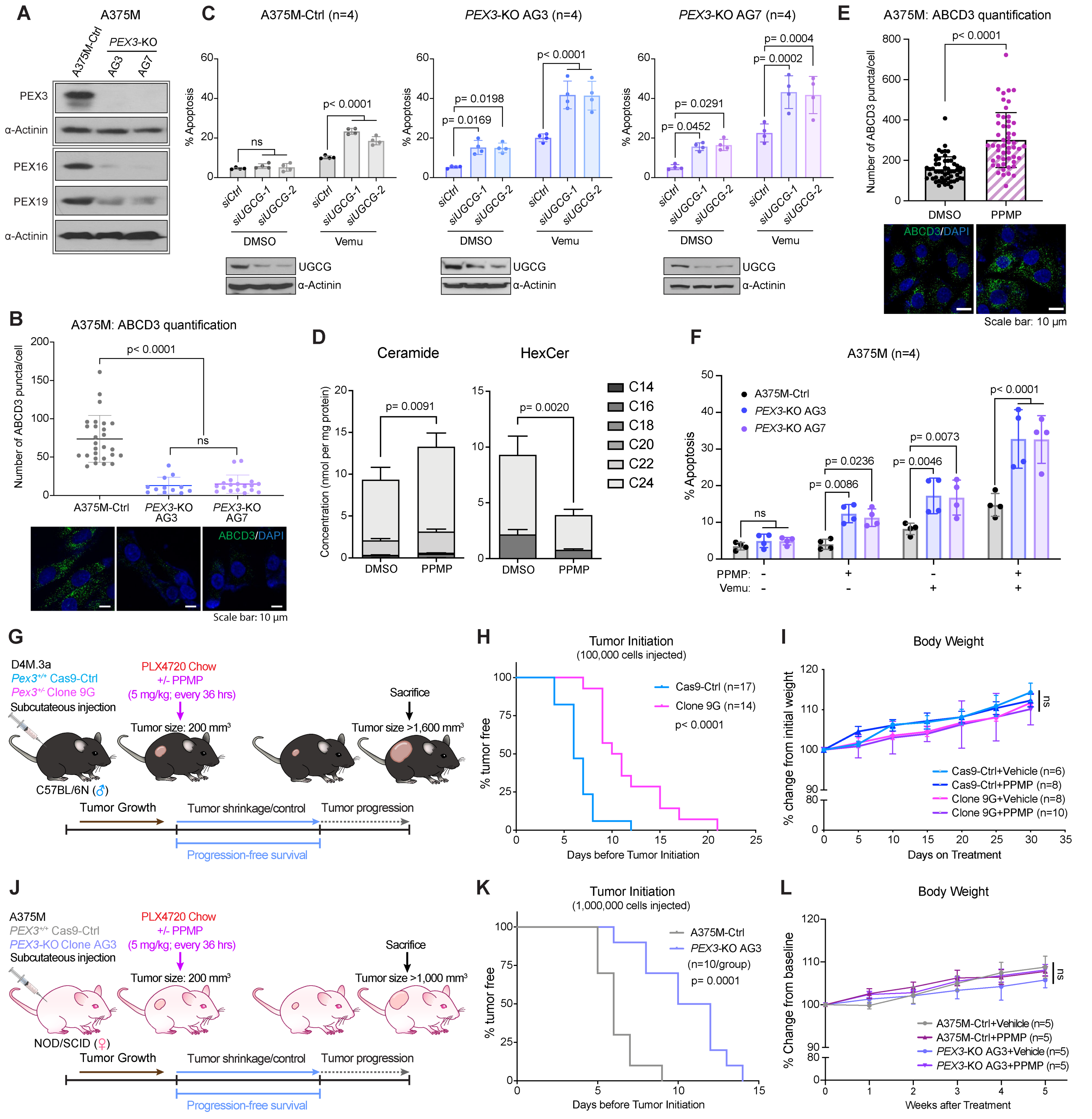

Figure S4. UGCG blockade increases vemu sensitivity in PEX3-deficient melanomas.
(A) Western blot analysis of the indicated proteins and **(B)** number of ABCD3 puncta in A375M Cas9-Ctrl (A375M-Ctrl), *PEX3*-KO (*PEX3*$^{-/-}$) Clone AG3 and AG7 cells. Representative IF staining for ABCD3 (green) and DAPI nuclear stain (blue) are presented (n=3). One-way ANOVA. **(C, F)** Percent apoptosis (PI^{+}/Annexin V^{+}, PI^{-}/Annexin V^{+}) detected in A375M-Ctrl, *PEX3*-KO AG3 and AG7 cells following **(C)** *UGCG* knockdown and treatment with vemu or DMSO control (n=4), or **(F)** PPMP treatment alone or in combination with vemu (n=3). **(D)** Concentrations of ceramides and HexCer detected in A375M cells treated with PPMP or DMSO for 24 hours (n=3). **(E)** Number of ABCD3 puncta in A375M cells treated with PPMP or DMSO for 72 hours. Representative IF staining for ABCD3 (green) and DAPI nuclear stain (blue) are presented (n=3). Two-sided unpaired t-test. **(C, D, F)** Two-way ANOVA. **(B-F)** Data shown as mean values, error bars represent SD. **(G, J)** Schematic of the experimental design, related to Figure 3. **(H, K)** Kaplan-Meier curves showing initiation of **(H)** D4M.3a Cas9-Ctrl- or 9G-derived melanomas (100,000 cells per mice injected), and **(K)** A375M-Ctrl or AG3 (*PEX3*-KO)-derived melanomas (1,000,000 cells per mice injected). Log-rank test. **(I, L)** Relative weight change (% initial body weight prior to treatment) of mice bearing **(I)** D4M.3a Cas9-Ctrl- or 9G-derived melanomas, or **(L)** A375M-Ctrl or AG3 (*PEX3*-KO)-derived melanomas after indicated treatment. Number of biological replicates (mice) is indicated in each graph. Two-way ANOVA. Data represent mean ± SEM.

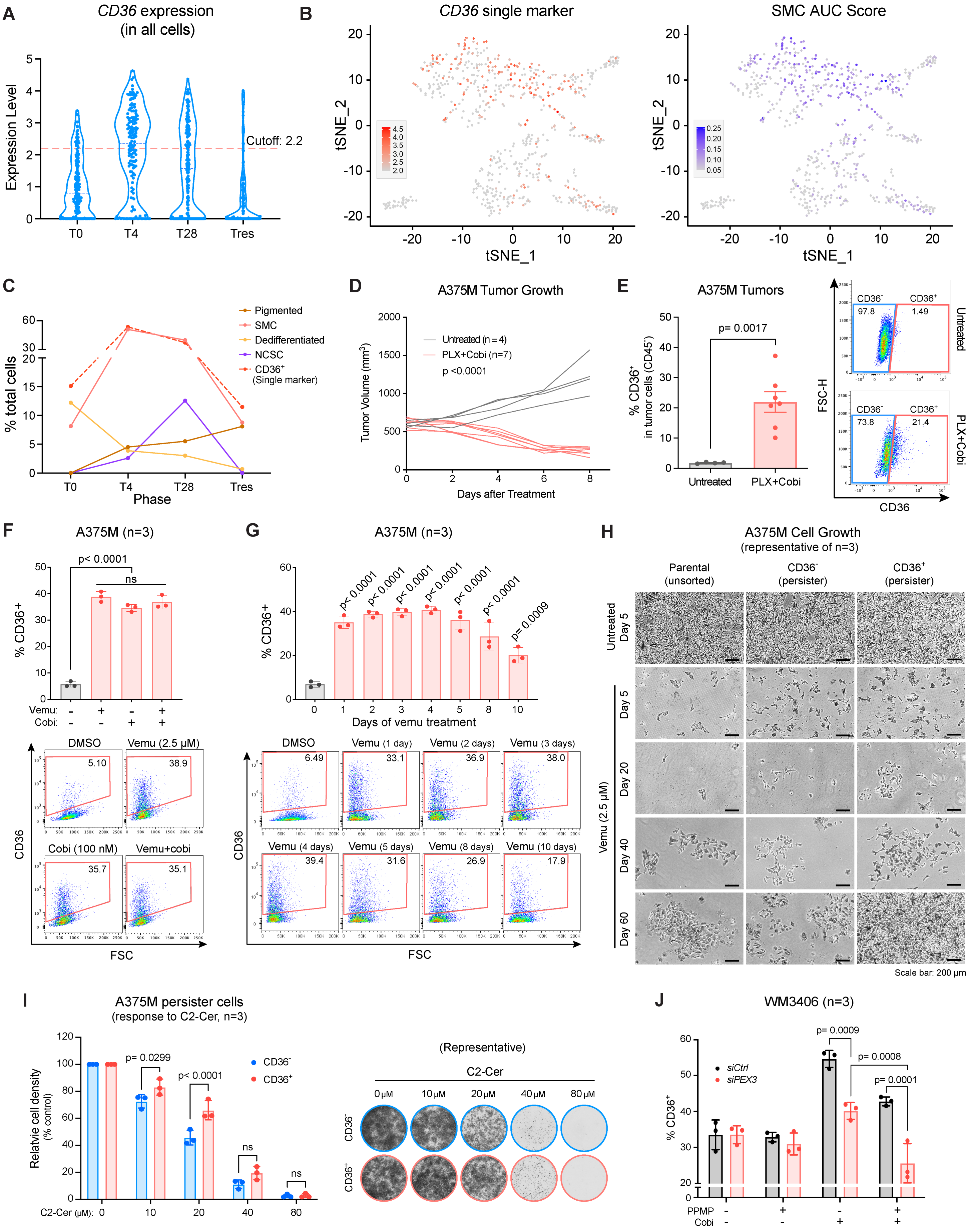
A
CD36 expression (in all cells)
Expression Level
Cutoff: 2.2
T0
T4
T28
Tres
B
CD36 single marker
SMC AUC Score
tSNE_2
tSNE_1
C
% total cells
Phase
Pigmented
SMC
Dedifferentiated
NCSC
CD36+ (Single marker)
D
A375M Tumor Growth
Tumor Volume (mm3)
Untreated (n = 4)
PLX+Cobi (n=7)
p <0.0001
Days after Treatment
E
A375M Tumors
% CD36+ in tumor cells (CD45-)
p= 0.0017
Untreated
PLX+Cobi
CD36-
CD36+
97.8
1.49
73.8
21.4
FSC-H
CD36
F
A375M (n=3)
% CD36+
p< 0.0001
ns
Vemu:
Cobi:
DMSO
Vemu (2.5 μM)
Cobi (100 nM)
Vemu+cobi
5.10
38.9
35.7
35.1
CD36
FSC
G
A375M (n=3)
% CD36+
p< 0.0001
p= 0.0009
Days of vemu treatment
DMSO
Vemu (1 day)
Vemu (2 days)
Vemu (3 days)
Vemu (4 days)
Vemu (5 days)
Vemu (8 days)
Vemu (10 days)
6.49
33.1
36.9
38.0
39.4
31.6
26.9
17.9
CD36
FSC
H
A375M Cell Growth
(representative of n=3)
Parental (unsorted)
CD36- (persister)
CD36+ (persister)
Untreated Day 5
Vemu (2.5 μM)
Day 5
Day 20
Day 40
Day 60
Scale bar: 200 μm
I
A375M persister cells
(response to C2-Cer, n=3)
Relatvie cell density (% control)
p= 0.0299
p< 0.0001
ns
CD36-
CD36+
C2-Cer (μM):
(Representative)
C2-Cer
0 μM
10 μM
20 μM
40 μM
80 μM
J
WM3406 (n=3)
% CD36+
siCtrl
siPEX3
p= 0.0009
p= 0.0008
p= 0.0001
PPMP
Cobi

Figure S5. CD36 marks a distinct population of melanoma persister cells.
(A) Violin plot of scRNA-seq data highlighting the distribution of *CD36* and the cutoff line set to distinguish $CD36^-$ (< 2.2) and $CD36^+$ (≥ 2.2) cells in different phases of MAPKi treatments. **(B)** A total of 674 melanoma cells (*Rambow 2018* dataset) were projected in a two-dimensional space by t-SNE, comparing the distribution of $CD36^+$ cells (left) defined by high *CD36* expression as a single marker (normalized gene expression ≥ 2.2, colored in red) versus SMCs (right) defined by AUCell analysis (SMC AUCell score ≥ 0.05, colored in purple). **(C)** Dynamics of the different melanoma cell states at the indicated time points. **(D)** Individual growth of A375M-derived melanomas treated with combined BRAF/MEK inhibitors (PLX4720+cobi), related to Figure 4E. **(E)** Percentage of $CD36^+$ cells in total ($CD45^-$) tumor cells, isolated from A375M-derived melanomas following PLX4720+cobi treatment for 8 days. Two-sided unpaired t-test. Data represent mean ± SEM. **(F, G)** Percentage of $CD36^+$ populations in A375M cells following **(F)** indicated MAPK inhibitors treatment for 48 hours, or **(G)** vemu treatment for indicated time. Representative flow cytometric images are presented below (n=3). One-way ANOVA. **(H)** Representative image of parental (unsorted), $CD36^-$ and $CD36^+$ persister A375M cells following vemu treatment for indicated days (representative of n=3). **(I)** Relative survival of $CD36^+$ versus $CD36^-$ persister A375M cells following C2-Cer treatment at indicated doses, measured by crystal violet staining. Representative images of crystal violet-stained cells are presented (n=3). **(H, I)** $CD36^-$ and $CD36^+$ persister A375M cells were sorted after 48-hour vemu treatment (see Figure 5A). **(J)** Percentage of $CD36^+$ populations in WM3406 cells following *PEX3* knockdown and the indicated treatment (n=3). **(D, I, J)** Two-way ANOVA. **(F, G, I, J)** Data are presented as mean ± SD.

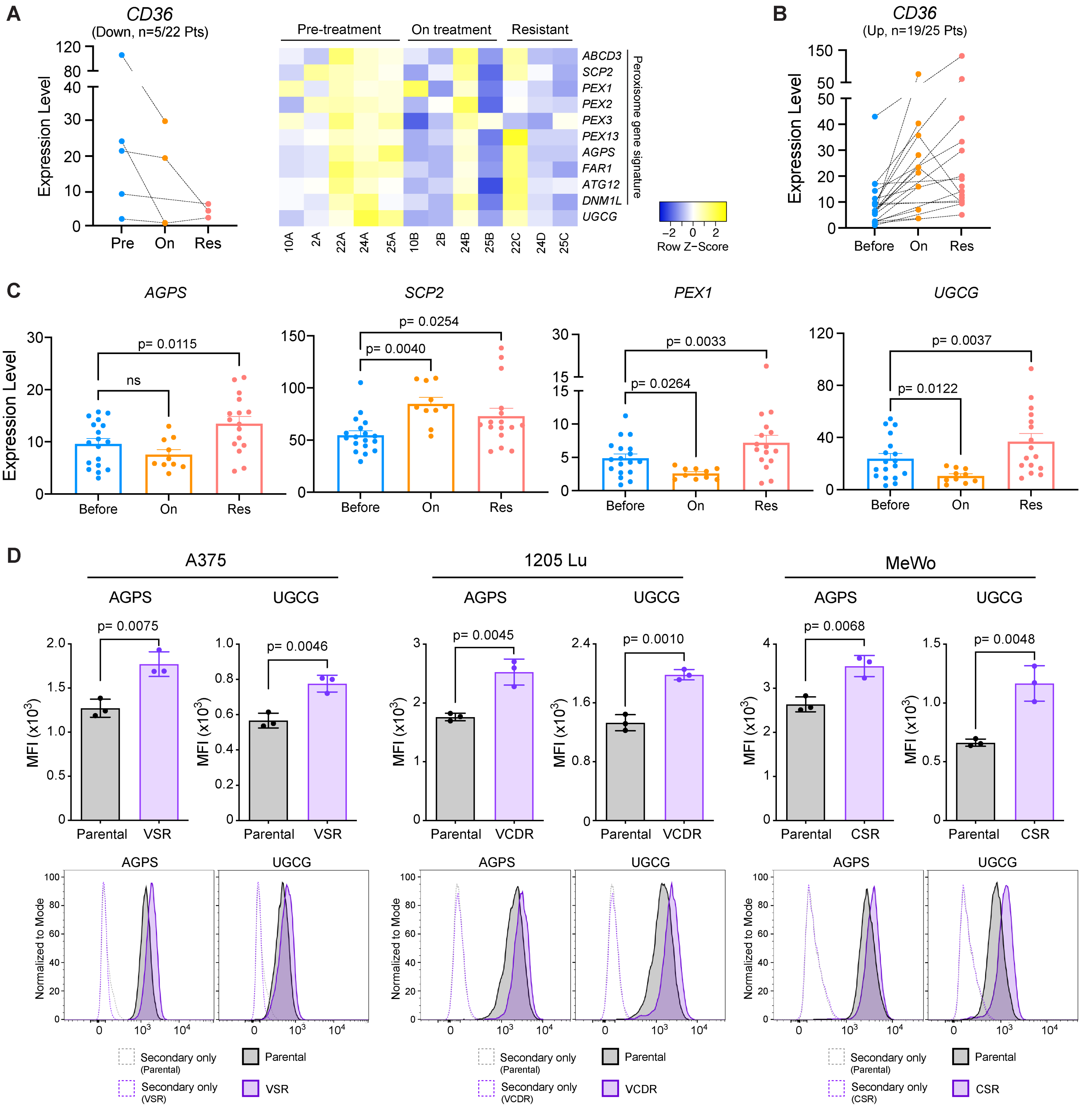

Figure S6. MAPKi-induced upregulation of *CD36* is associated with increased peroxisome and UGCG activity in therapy-resistant melanomas.
(A) Expression of *CD36* (left) and relative expression of a peroxisomal gene signature and *UGCG* (right) in a cohort of melanoma samples collected pre-, on-, or relapsed on MAPK-targeted therapy. n=5 out of a total of 22 patients (*Kwong 2015* dataset), with an overall trend of *CD36* downregulation following MAPKi treatment. **(B)** Expression of *CD36* and **(C)** expressions of *AGPS*, *SCP2*, *PEX1* and *UGCG* in a cohort of melanoma samples (n=19 out of a total of 25 patients, *Hugo 2015* and *Song 2017* datasets) collected pre-, on-, or relapsed on MAPK-targeted therapy showing an overall trend of *CD36* induction upon MAPKi. Data are presented as Mean + SEM. One-way ANOVA. **(D)** Relative expressions of AGPS and UGCG in a panel of parental versus MAPKi-resistant melanoma cell lines (see Table S1). Representative flow cytometric histograms are presented below (n=3). Two-sided unpaired t-test. Data are presented as Mean ± SD.

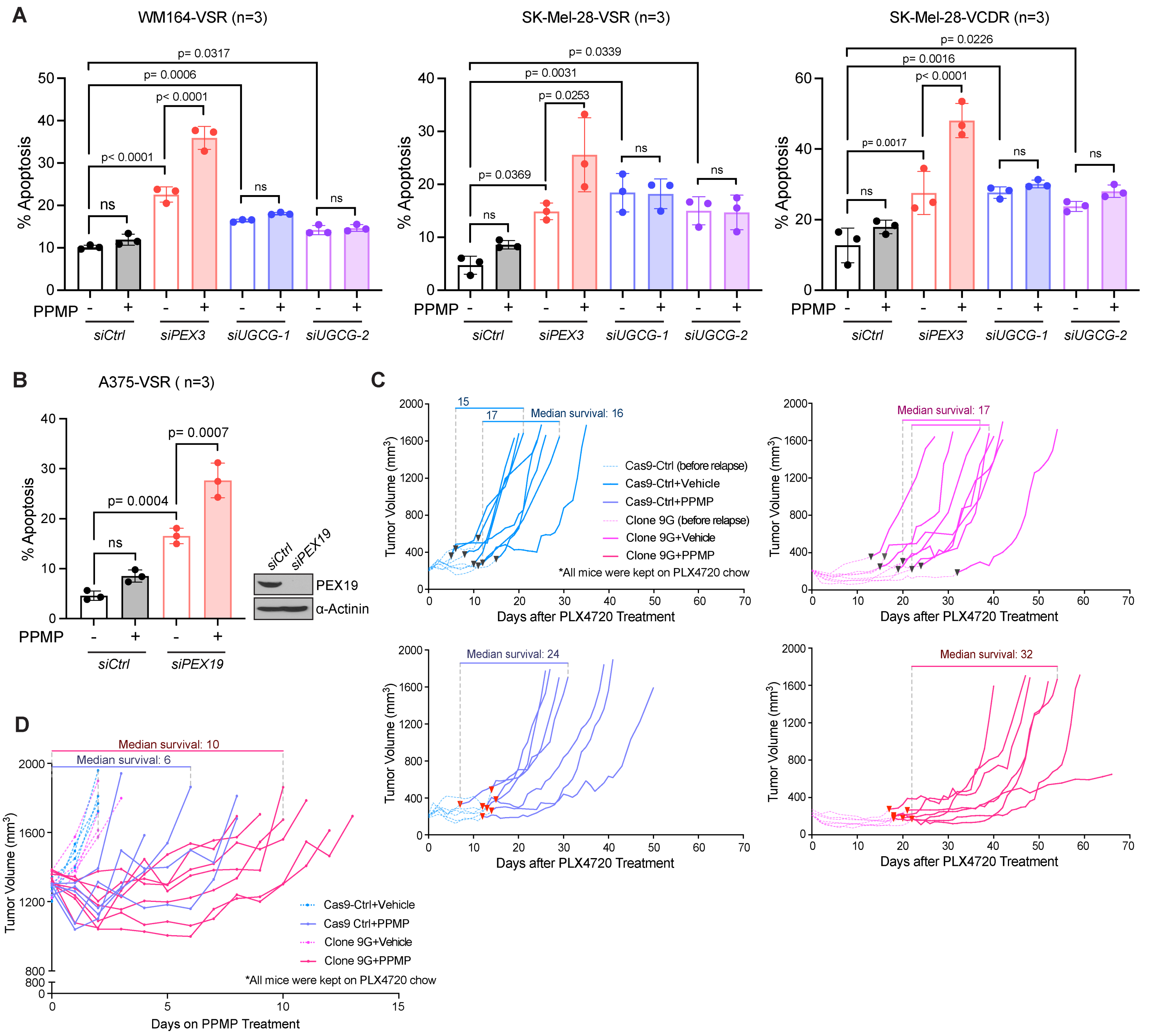

Figure S7. MAPKi-resistant melanomas are sensitive to combined inhibition of peroxisomes and UGCG.
(A, B) Percent apoptosis (PI+/Annexin V+, PI-/Annexin V+) detected in **(A)** a panel of MAPKi-resistant melanoma cells following *PEX3* or *UGCG* knockdown, or **(B)** A375-VSR cells following *PEX19* knockdown. Cells were maintained in the presence of indicated MAPK inhibitors and were treated with PPMP or equal volume of DMSO as control (Detailed treatment and timeline are presented in Table S1). Data represent mean ± SD (n=3). Two-way ANOVA. **(C, D)** Individual growth of D4M.3a Cas9-Ctrl- or 9G-derived melanomas in mice receiving indicated treatment, related to Figure 7C and 7E, respectively. Mice were treated with PPMP or vehicle **(C)** after tumors relapsed on PLX4720, annotated with red or grey triangles, or **(D)** after relapsed tumor reached a volume of 1,300 mm³. Median survival is indicated in each graph. All mice were kept on PLX4720 chow after PLX4720 treatment initiated when individual tumor first reached a volume of 200 mm³. Number of biological replicates (mice) is indicated in each graph.

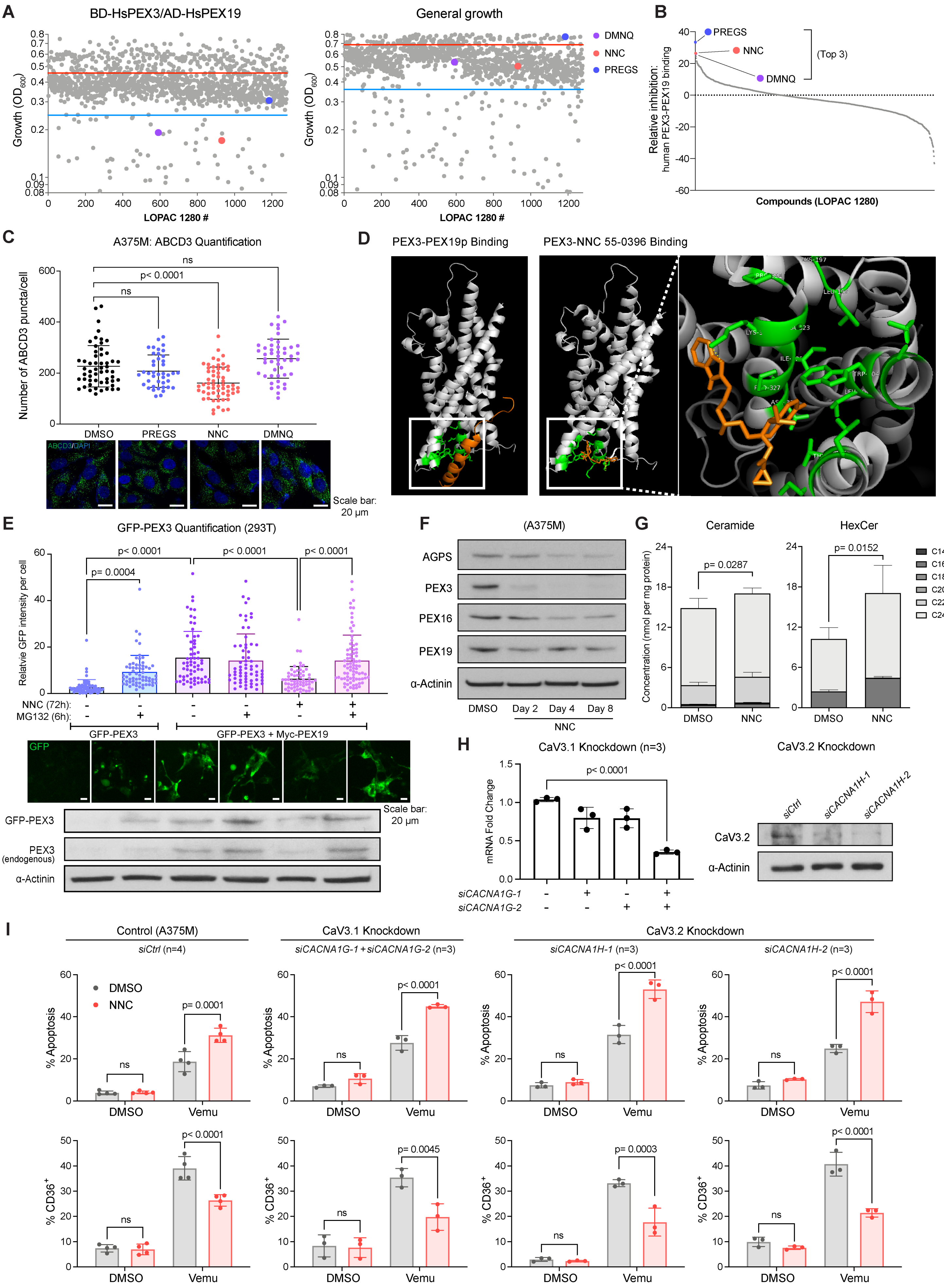

A
BD-HsPEX3/AD-HsPEX19
General growth
Growth (OD600)
LOPAC 1280 #
DMNQ
NNC
PREGS
B
Relative inhibition: human PEX3-PEX19 binding
Compounds (LOPAC 1280)
(Top 3)
C
A375M: ABCD3 Quantification
Number of ABCD3 puncta/cell
ns
p< 0.0001
DMSO
PREGS
NNC
DMNQ
ABCD3/DAPI
Scale bar: 20 μm
D
PEX3-PEX19p Binding
PEX3-NNC 55-0396 Binding
E
GFP-PEX3 Quantification (293T)
Relatvie GFP intensity per cell
p= 0.0004
p< 0.0001
NNC (72h):
MG132 (6h):
GFP-PEX3
GFP-PEX3 + Myc-PEX19
GFP
Scale bar: 20 μm
GFP-PEX3
PEX3 (endogenous)
α-Actinin
F
(A375M)
AGPS
PEX3
PEX16
PEX19
α-Actinin
DMSO
Day 2
Day 4
Day 8
NNC
G
Ceramide
HexCer
Concentration (nmol per mg protein)
p= 0.0287
p= 0.0152
C14
C16
C18
C20
C22
C24
H
CaV3.1 Knockdown (n=3)
mRNA Fold Change
p< 0.0001
siCACNA1G-1
siCACNA1G-2
CaV3.2 Knockdown
siCtrl
siCACNA1H-1
siCACNA1H-2
CaV3.2
α-Actinin
I
Control (A375M)
siCtrl (n=4)
CaV3.1 Knockdown
siCACNA1G-1 + siCACNA1G-2 (n=3)
CaV3.2 Knockdown
siCACNA1H-1 (n=3)
siCACNA1H-2 (n=3)
% Apoptosis
% CD36+
DMSO
NNC
Vemu
p= 0.0001
p= 0.0045
p= 0.0003

Figure S8. Identifying NNC 55-0396 as a PEX3-PEX19 binding inhibitor.

(A) Growth of HF7c *pdr5Δ* cells expressing human BD-PEX3/AD-PEX19 fusion proteins (left) or the parental pGAD424 and pGBT9 two-hybrid vectors (right) in the presence of compounds (100μM) from the LOPAC 1280 library. Each dot represents relative growth of yeast measured by OD_{600} in the presence of an individual compound. The red line marks the average growth level and the blue line indicates a growth reduction of 1.5 times the standard deviation. **(B)** Relative inhibition of human PEX3-PEX19 binding by compounds from the LOPAC 1280 library. Each dot represents percent inhibition of the BD-PEX3/AD-PEX19-expressing yeast grown in the presence of an individual compound, subtracted the percent inhibition of the same compound on general growth of the pGAD424/pGBT9-expressing yeast (See Supplemental Methods). Top three hits with the highest activity against human PEX3-PEX19 binding, pregnenolone sulfate (PREGS), NNC 55-0396 (NNC), and 2,3-Dimethoxy-1,4-naphthoquinone (DMNQ), are highlighted. **(A, B)** Figures were generated from the *Banerjee 2021* yeast two-hybrid drug screen data (see Supplemental Methods). **(C)** Number of ABCD3 puncta in A375M cells treated with DMSO control, PREGS (10μM), NNC (4μM), or DMNQ (5μM) for 72 hours. Representative IF staining for ABCD3 (green) and DAPI nuclear stain (blue) are presented (data pooled from n=3 independent experiments). One-way ANOVA. **(D)** Structural analysis showing human PEX19-binding site on PEX3 (left) and predicted binding site of NNC on human PEX3 protein in close proximity to the PEX19-binding site (right). Figures were generated using AutoDock. **(E)** Relative expression of GFP-PEX3 measured by GFP MFI (top) or GFP-PEX3 and endogenous PEX3 levels assessed by western blot (bottom) in 293T cells overexpressing GFP-PEX3 alone or 293T cells co-transfected with GFP-PEX3- and Myc-PEX19-expressing vectors, following indicated treatment (n=3). **(F)** Western blot analysis of a panel of peroxisomal proteins in A375M cells following NNC (4μM) treatment for indicated time, representative of n=3. **(G)** Concentrations of ceramides and HexCer in A375M cells following NNC (4μM) treatment for 10 days (n=3). **(H)** qPCR (left, n=3) and western blot analysis (right, n=2) to confirm knockdown efficiency of *CACNA1G* (encodes CaV3.1) and *CACNA1H* (encodes CaV3.2), respectively. Note that *siCACNA1G-1* and *siCACNA1G-2* were co-transfected in A375M cells to effectively knockdown *CACNA1G*. One-way ANOVA. **(I)** Percent apoptosis (top) and percentage of $CD36^{+}$ populations (bottom) detected in A375M cells following siRNA-mediated knockdown of *CACNA1G* or *CACNA1H* and subsequent treatment with vemu for 24 hours (n=4 for the *siCtrl* group, n=3 for all CaV3.1 and CaV3.2 knockdown conditions). **(E, G, I)** Two-way ANOVA. **(C, E, G-I)** Data shown as mean values, error bars represent SD.

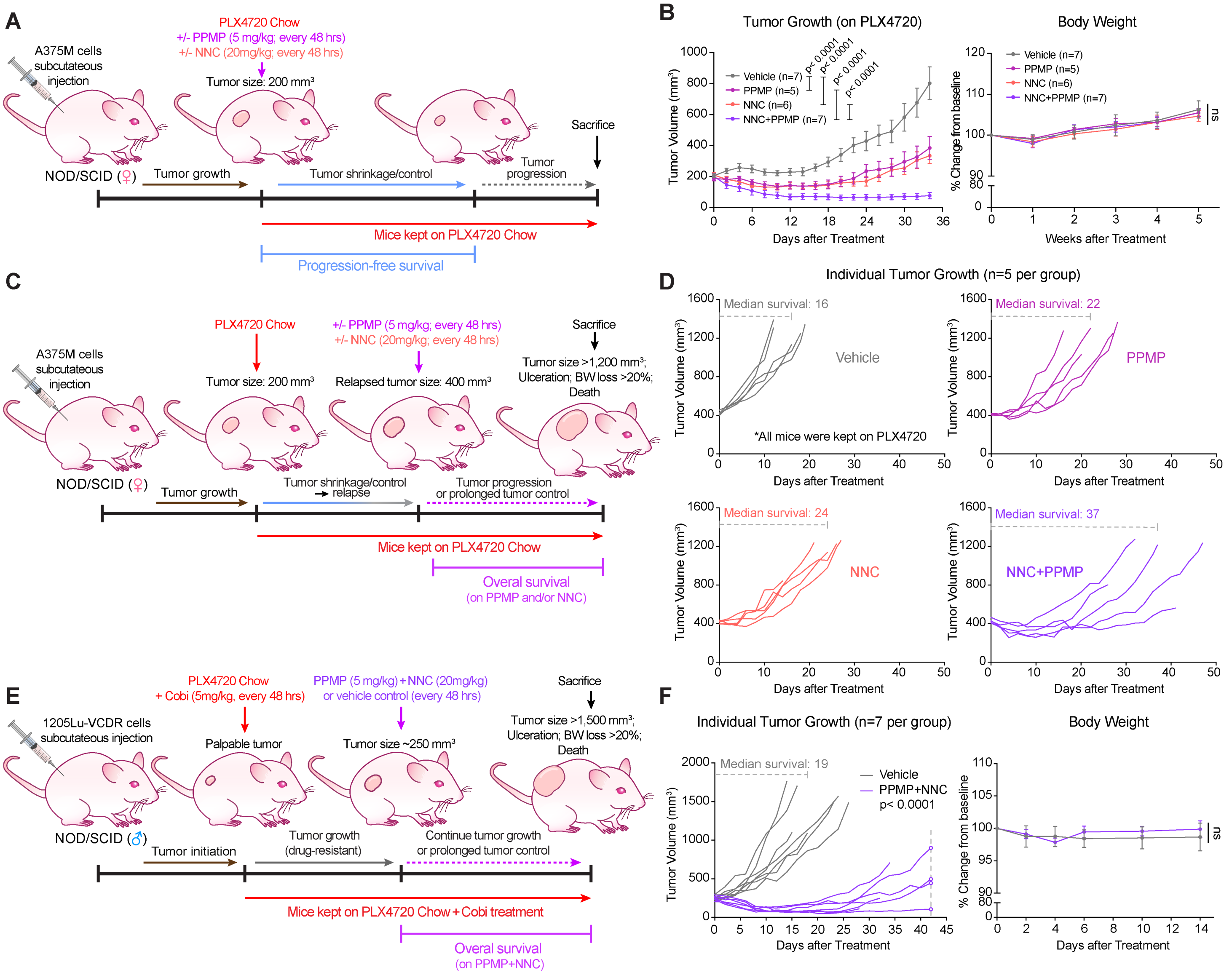

Figure S9. PEX3-PEX19 binding inhibitor NNC 55-0396 demonstrates anti-tumoral activity in preclinical melanoma models.

(A, C, E) Schematics of the experimental design, related to Figure 8F-8H, 8I, and 9F, respectively. **(B)** Tumor growth curve (left, related to Figure 8F-8H) and relative weight change (right, % initial body weight prior to treatment) of mice bearing A375M-derived melanomas receiving indicated treatments. **(D)** Individual growth of A375M-derived melanomas in mouse receiving indicated treatments, related to Figure 8I. Mice were treated with vehicle, NNC, PPMP, or NNC+PPMP after relapsed (PLX4720-resistant) tumor reached a volume of 400 mm³. Median survival is indicated in each graph. All mice were kept on PLX4720 chow after PLX4720 treatment initiated when individual tumor first reached a volume of 200 mm³. **(F)** Individual tumor growth curve (left) and percent weight change (right) of mice bearing 1205Lu-VCDR-derived melanomas receiving indicated treatments. All mice were treated with PLX4720+cobi throughout the experiment. Vehicle or NNC+PPMP treatments were initiated once PLX4720/cobi dual-resistant tumors reached a volume of approximately 250 mm³. Number of biological replicates (mice) is indicated in each graph. **(B, F)** Two-way ANOVA. Data shown as mean values, error bars represent SEM.

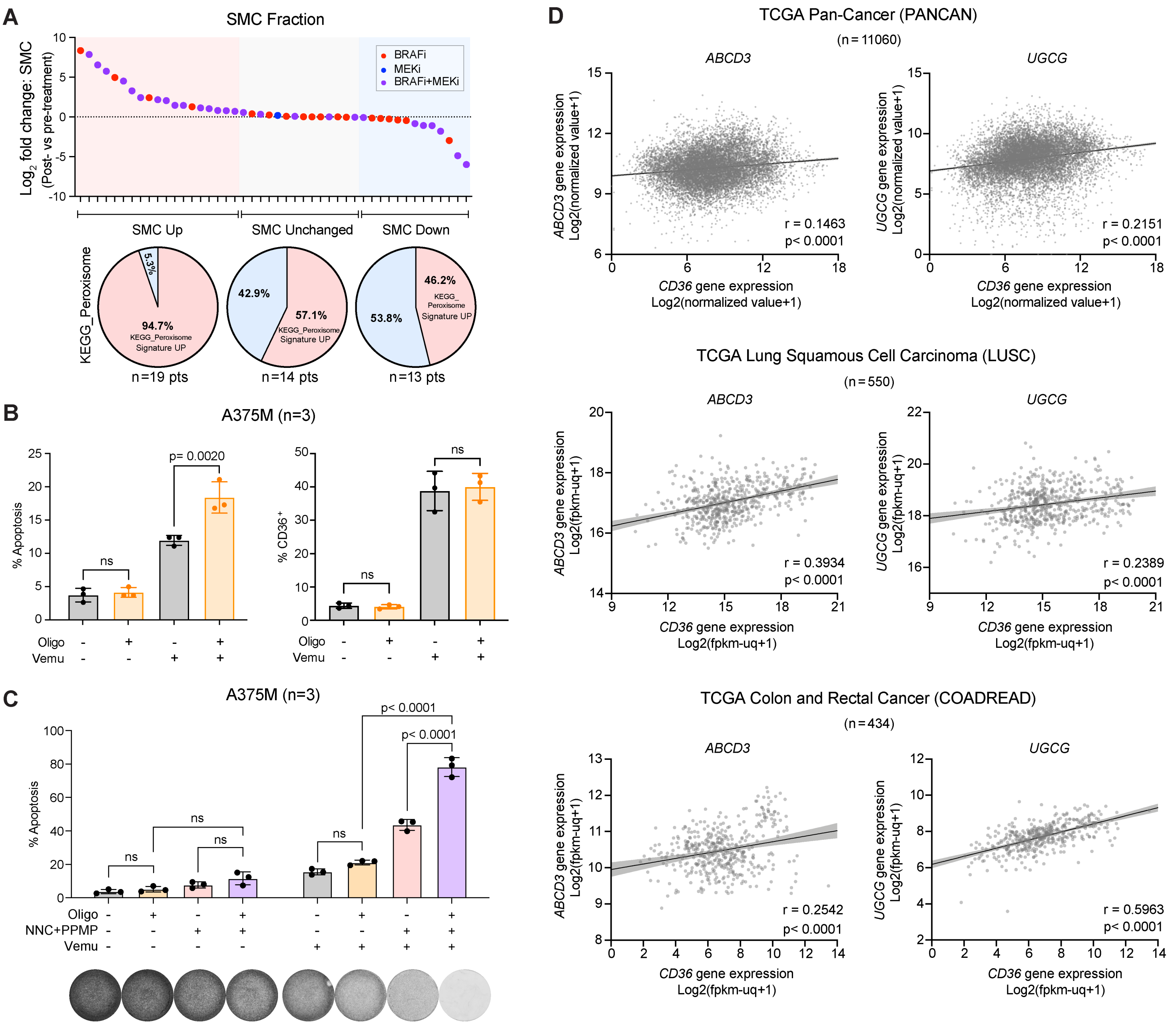

Figure S10. Perspectives on targeting peroxisomes and UGCG in CD36-expressing cancer cells.

(A) Top: Log_2 fold change of SMC abundance in samples from each patient collected post- versus pre-treatment with indicated MAPK inhibitors. A 1.5-fold increase is considered as "SMC up", and a 5% decrease is considered as "SMC down". Bottom: Pie charts showing percentage of patients in each group with increased or decreased transcript levels of peroxisome-related genes (KEGG_Peroxisome), related to Figure 1A. Total n=46 patients. **(B)** Percent apoptosis (left) and percentage of $CD36^+$ populations (right) detected in A375M cells following indicated treatment for 24 hours (n=3). **(C)** Percent apoptosis (top) and representative image of crystal violet-stained A375M cells (bottom) following indicated treatment for 24 hours (n=3). **(B, C)** Two-way ANOVA. Data presented as mean ± SD. **(D)** Correlation of *CD36* expression with gene expressions of *ABCD3* and *UGCG* in the TCGA Pan-cancer (PANCAN) dataset (top, n=11060), the TCGA Lung Squamous Cell Carcinoma (LUSC) dataset (middle, n=550), and the TCGA Colon and Rectal Cancer (COADREAD) dataset (bottom, n=434). Pearson rank-order. Solid lines show linear regression and shaded areas represent 95% confidence intervals of the linear regression.

A

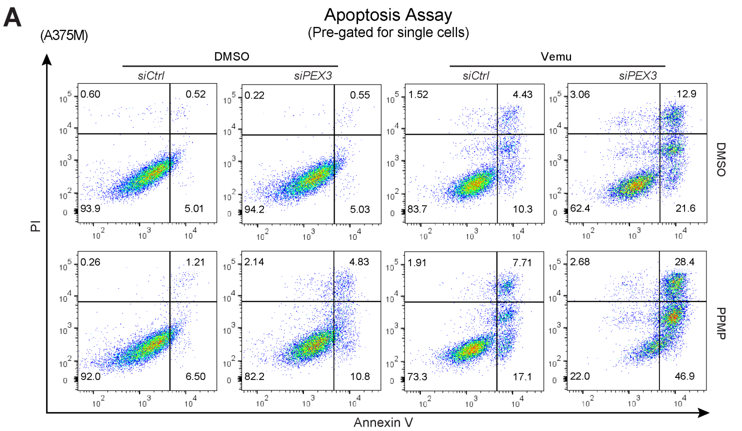

B

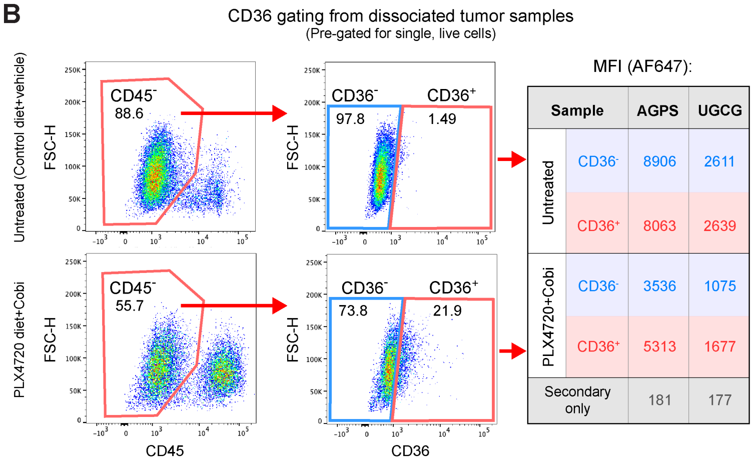

MFI (AF647):

Sample		AGPS	UCGG
Untreated	CD36⁻	8906	2611
	CD36⁺	8063	2639
PLX4720+Cobi	CD36⁻	3536	1075
	CD36⁺	5313	1677
Secondary only		181	177

C

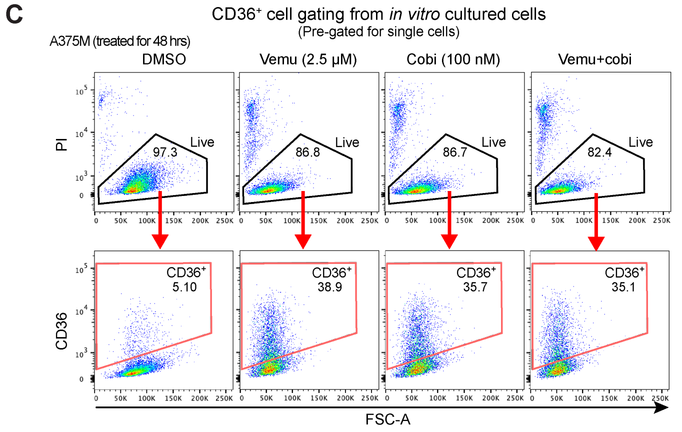

D

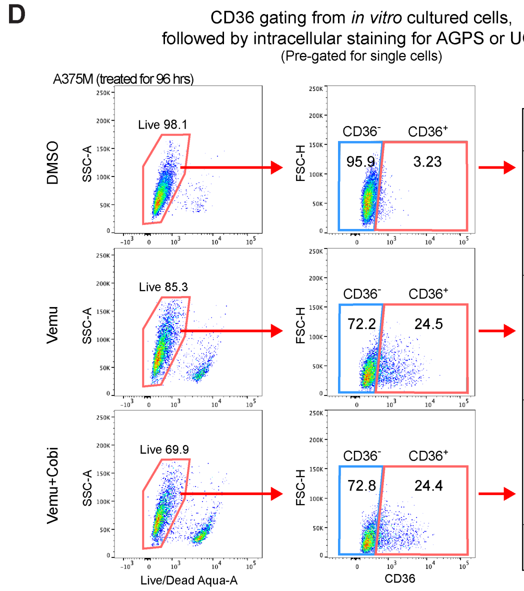

MFI (AF647):

Sample		AGPS	UCGG
DMSO	CD36⁻	2715	1403
	CD36⁺	2860	1461
Vemu	CD36⁻	1741	986
	CD36⁺	2226	1283
Vemu+Cobi	CD36⁻	1529	873
	CD36⁺	1981	1156
Secondary only		112	228

Panel A: Apoptosis Assay

Antibody/dye	Dilution
Alexa Fluor™ 647-Annexin V (Invitrogen™, A23204)	1:1000
Propidium Iodide (PI) (BD Biosciences, 556463)	1:250

Panel B: Staining of tumor samples

	Antibody/dye	Dilution
Panel B1 (to assess AGPS MFI)	Aqua Fixable Viability dye (Invitrogen, L34957)	1:200
	Brilliant Violet 421™ anti-human CD36, Clone 5-271 (BioLegend, 336229)	1:400
	BUV395-CD45, Clone 30-F11 (BD Bioscience, 564279)	1:400
	Unconjugated anti-AGPS primary antibody (Abcam, ab236621)	1:400
	Alexa Fluor™ 647 donkey anti-rabbit secondary antibody (Invitrogen™, A31573)	1:2000
Panel B2 (to assess UGCG MFI)	Aqua Fixable Viability dye (Invitrogen, L34957)	1:200
	Brilliant Violet 421™ anti-human CD36, Clone 5-271 (BioLegend, 336229)	1:400
	BUV395-CD45, Clone 30-F11 (BD Bioscience, 564279)	1:400
	Unconjugated anti-UGCG primary antibody (Proteintech, 12869-1-AP)	1:200
	Alexa Fluor™ 647 donkey anti-rabbit secondary antibody (Invitrogen™, A31573)	1:2000

Panel C: CD36 Staining (*in vitro*)

Antibody/dye	Dilution
Propidium Iodide (PI) (BD Biosciences, 556463)	1:250
Brilliant Violet 421™ anti-human CD36, Clone 5-271 (BioLegend, 336229)	1:400

Panel D: CD36+UGCG Staining (*in vitro*)

	Antibody/dye	Dilution
Panel D1 (to assess AGPS MFI)	Aqua Fixable Viability dye (Invitrogen, L34957)	1:200
	Brilliant Violet 421™ anti-human CD36, Clone 5-271 (BioLegend, 336229)	1:400
	Unconjugated anti-AGPS primary antibody (Abcam, ab236621)	1:800
	Alexa Fluor™ 647 donkey anti-rabbit secondary antibody (Invitrogen™, A31573)	1:2000
Panel D2 (to assess UGCG MFI)	Aqua Fixable Viability dye (Invitrogen, L34957)	1:200
	Brilliant Violet 421™ anti-human CD36, Clone 5-271 (BioLegend, 336229)	1:400
	Unconjugated anti-UGCG primary antibody (Proteintech, 12869-1-AP)	1:200
	Alexa Fluor™ 647 donkey anti-rabbit secondary antibody (Invitrogen™, A31573)	1:2000

Figure S11. Gating strategies and detailed panels of antibodies/dyes used for flow cytometry. (A) Gating strategy for apoptosis assay. Percent apoptotic cells are calculated by the sum of PI/Annexin V double-positive and Annexin V-positive staining. **(B)** Gating strategy and flow panel related to Figure 4F and S5E. **(C)** Gating strategy for cultured CD36$^-$ and CD36$^+$ melanoma cells. **(D)** Gating strategy and flow panel related to Figure 4I.

Supplemental Table 1. Detailed information of human cell lines, source, culture conditions, treatment, and experimental timeline.

Cell line & source	Cultured in	Treatment	Timeline
A375M<br>Gifted by Dr. Ghanem Ghanem (Institut Jules Bordet)	DMEM	Vemu (2.5 μM)	Day 1: seed + siRNA transfection<br>Day 3: treatment<br>Day 4: harvest (24h treatment)
		Vemu (2.5 μM)<br>PPMP (7.5 μM)	Day 1: seed + siRNA transfection<br>Day 3: treatment<br>Day 4: harvest (24h treatment)
		Cobi (100 nM)	Added at the same time with vemu
		Vor (1 μM)	Added at the same time with vemu
		Oligo (1 μM)	Added at the same time with vemu
1205Lu<br>Purchased from Rockland Immunochemicals (The Wistar Institute)	RPMI	Vemu (5 μM)<br>Cobi (50 nM)	Day 1: seed + siRNA transfection<br>Day 3: treatment<br>Day 4: harvest (24h treatment)
		Vemu (5 μM)<br>Cobi (50 nM)<br>PPMP (10 μM)	Day 1: seed + siRNA transfection<br>Day 3: treatment<br>Day 4: harvest (24h treatment)
WM3406<br>Gifted by Dr. April Rose (Lady Davis Institute)	RPMI<br>1x GlutaMax	Cobi (100 nM)	Day 1: seed + siRNA transfection<br>Day 2: treatment<br>Day 4: harvest (48h treatment)
		Cobi (100 nM)<br>PPMP (5 μM)	Day 1: seed + siRNA transfection<br>Day 2: treatment<br>Day 4: harvest (48h treatment)
MeWo<br>Gifted by Dr. Ian R Watson (MUHC Research Institute)	DMEM	Cobi (100 nM)	Day 1: seed + siRNA transfection<br>Day 2: treatment<br>Day 4: harvest (48h treatment)
		Cobi (100 nM)<br>PPMP (5 μM)	Day 1: seed + siRNA transfection<br>Day 2: treatment<br>Day 4: harvest (48h treatment)
MelST<br>Gifted by Dr. Robert Weinberg (Whitehead Institute)	DMEM	Vemu (5 μM)<br>Cobi (100 nM)	Day 1: seed + siRNA transfection<br>Day 2: treatment<br>Day 4: harvest (48h treatment)
A375-VSR<br>Gifted by Dr. Gideon Bollag (Plexxikon)	DMEM<br>Vemu (2.5 μM)	Vemu (2.5 μM)<br>PPMP (7.5 μM)	Day 1: seed + siRNA transfection<br>Day 2: treatment<br>Day 4: harvest (48h treatment)
1205Lu-VCDR<br>Generated in this study	RPMI<br>Vemu (5 μM)<br>Cobi (50 nM)	Vemu (5 μM)<br>Cobi (50 nM)<br>PPMP (5 μM)	Day 1: seed + siRNA transfection<br>Day 2: treatment<br>Day 4: harvest (48h treatment)
WM164-VSR<br>(First described in PMID: 33690225)	DMEM<br>Vemu (1 μM)	Vemu (1 μM)<br>PPMP (10 μM)	Day 1: seed + siRNA transfection<br>Day 2: treatment<br>Day 4: harvest (48h treatment)
SK-Mel-28-VSR<br>(First described in PMID: 33690225)	DMEM<br>Vemu (2.5 μM)	Vemu (2.5 μM)<br>PPMP (10 μM)	Day 1: seed + siRNA transfection<br>Day 2: treatment<br>Day 4: harvest (48h treatment)
SK-Mel-28-VCDR<br>Generated in this study	DMEM<br>Vemu (2.5 μM)<br>Cobi (100 nM)	Vemu (2.5 μM)<br>Cobi (100 nM)<br>PPMP (5 μM)	Day 1: seed + siRNA transfection<br>Day 2: treatment<br>Day 4: harvest (48h treatment)
MeWo-CSR<br>Generated in this study	DMEM<br>Cobi (400 nM)	Cobi (400 nM)<br>PPMP (5μM)	Day 1: seed + siRNA transfection<br>Day 2: add Cobi<br>Day 3: add PPMP<br>Day 5: harvest (72h Cobi+48h PPMP)

* All cells were cultured in the indicated medium supplemented with 10% FBS and 1x Pen/Strep.

Supplemental Table 2. siRNAs.

Gene name	siRNA	Duplex Sequence (5'-3')
PEX3 (human)	*siPEX3*	rCrGrGrArCrArGrArUrCrCrArUrUrCrArGrUrUrGrCrAGT
		rArCrUrGrCrArArArCrUrGrArArUrGrGrArUrCrUrGrUrCrCrGrUrUr
	siPEX3-2	rGrArUrCrUrGrArArGrArUAArUrArArGrUrUrUrCrArCAA
		rUrUrGrUrGrArArArCrUrUrArUrUrArUrCrUrUrCrArGrArUrCrCrU
PEX19 (human)	*siPEX19*	rGrUrGrArArCrArGrUrGrUrCrUrGrArUrCrArUrGrUrGrAAA
		rUrUrUrCrArCrArUrGrArUrCrArGrArCrArCrUrGrUrUrCrArCrCrA
UGCG (human)	*siUGCG-1*	rCrUrUrCrArCrArUrCrCrArArGrArUrArCrUrArUrArUrCTC
		rGrArGrArUrArUrArGrUrArUrCrUrUrGrGrArUrGrUrGrArArGrUrU
	siUGCG-2	rGrCrUrUrUrGrUrGrArCrUrGrUrArUrArUrArArArGrGrAAA
		rUrUrUrCrCrUrUrUrArUrArUrArCrArGrUrCrArCrArArArGrCrUrG
CACNA1G (human)	*siCACNA1G-1*	rGrArGrCrUrUrArCrCrArArCrGrCrCrCrUrArGrArArArUCA
		rUrGrArUrUrUrCrUrArGrGrGrCrGrUrUrGrGrUrArArGrCrUrCrCrU
	siCACNA1G-2	rGrGrUrCrCrCrUrUrUrGrGrCrUrArCrArUrCrArArGrArATC
		rGrArUrUrCrUrUrGrArUrGrUrArGrCrCrArArArGrGrGrArCrCrArU
CACNA1H (human)	*siCACNA1H-1*	rGrGrUrUrArArArUrGrUrUrGrCrArArUrArArUrCrUrGrATG
		rCrArUrCrArGrArUrUrArUrUrGrCrArArCrArUrUrUrArArCrCrUrG
	siCACNA1H-2	rGrUrArArUrCrArUrGrCrUrCrArArCrUrGrCrGrUrGrArCCC
		rGrGrGrUrCrArCrGrCrArGrUrUrGrArGrCrArUrGrArUrUrArCrCrA
Ugcg (mouse)	*siUgcg-1*	rArGrArArUrGrUrArArUrUrUrCrArUrGrArUrArCrArArGTA
		rUrArCrUrUrGrUrArUrCrArUrGrArArArUrUrArCrArUrUrCrUrUrA
	siUgcg-2	rGrUrArCrArUrUrGrCrUrGrArArGrArUrUrArCrUrUrUrATG
		rCrArUrArArArGrUrArArUrCrUrUrCrArGrCrArArUrGrUrArCrUrG
Gba (mouse)	*siGba-1*	rGrGrUrUrCrCrArArGrArGrCrUrArUrGrArUrArUrCrUrGTC
		rGrArCrArGrArUrArUrCrArUrArGrCrUrCrUrUrGrGrArArCrCrGrA
	siGba-2	rGrUrGrArArGrCrUrArCrUrCrArUrGrCrUrArGrArUrGrACC
		rGrGrUrCrArUrCrUrArGrCrArUrGrArGrUrArGrCrUrUrCrArCrArU
Negative control	*siCtrl*	N/A (Proprietary, AllStar Neg. Control siRNA, QIAGEN #1027281)

Supplemental Table 3. Detailed information of the primary antibodies used for western blotting, immunohistochemistry, and immunofluorescence staining.

**Target**	**Antibody full name**	**Source & Catalog #**	**Experiment**
PEX3	Anti-PEX3 antibody produced in rabbit	Sigma-Aldrich, HPA042830	WB, IF (human)
PEX3	PEX3 Polyclonal Antibody	ThermoFisher, PA5-115740	WB (murine)
PEX19	PEX19 Polyclonal antibody	Proteintech, 14713-1-AP	WB
PEX16	PEX16 Polyclonal antibody	Proteintech, 14816-1-AP	WB
UGCG	UGCG Antibody (1E5)	Novus Biologicals, H00007357-M03	WB
UGCG	UGCG Polyclonal antibody	Proteintech, 12869-1-AP	Flow, IF
AGPS	Anti-AGPS antibody	Abcam, ab236621	WB, Flow
CD36	Brilliant Violet 421™ anti-human CD36 Antibody	BioLegend, 336229	Flow, IF
ABCD3	Anti-PMP70 antibody [CL2524]	Abcam, ab211533	IF
ABCD3	Rabbit polyclonal anti-PMP70 antibody	Abcam, ab3421	IF
Myc-Tag	Myc-Tag (9B11) Mouse mAb	Cell Signaling #2276	IP
CaV3.1	CaV3.1 Polyclonal Antibody	ThermoFisher, PA5-37236	WB
CaV3.2	CaV3.2 Polyclonal Antibody	ThermoFisher, PA5-120102	WB
GAPDH	GAPDH (14C10) Rabbit mAb	Cell Signaling #2118	WB
α-Actinin	Anti-α-actinin Antibody (H-2)	Santa Cruz, sc-17829	WB
β-Actin	Monoclonal Anti-β-Actin antibody (clone AC-15)	Sigma-Aldrich #A5441	WB

Supplemental Table 4. RT-qPCR primers.

Gene name	Primer	Sequence (5'-3')
AGPS	Fwd	GTGACCCACTGACCGTATTT
	Rev	GCCATTGCTTCCGTAACTTG
PEX1	Fwd	CCTGTGTGCTACAAGTAGTCTG
	Rev	GGAATCCAGACTTTCCCAAGA
***SCP2* (N terminal)**	Fwd	AACTGTGCTACTGGTTCTACTG
	Rev	GGCTTCCCTTACTCATCTTCTC
UGCG	Fwd	GGATCAAGCAGGAGGACTTATAG
	Rev	CTTGAGTGGACATTGCAAACC
CACNA1H	Fwd	CAAGGATGGATGGGTGAACA
	Rev	GATGAGCAGGAAGGAGATGAAG
RPLP0	Fwd	TCCTCGTGGAAGTGACATCGT
	Rev	CTGTCTTCCCTGGGCATCA
ACTB	Fwd	AGGCACCAGGGCGTGAT
	Rev	GCCCACATAGGAATCCTTCTGAC

Supplemental Methods

Mice

For melanoma cell inoculation, D4M.3a Cas9-Ctrl, *Pex3*$^{+/-}$ 6D, and *Pex3*$^{+/-}$ 9G cells were injected to male C57BL/6N mice at 30,000 cells/mouse or 100,000 cells/mouse; A375M cells, A375M Cas9-Ctrl (A375M-Ctrl) and *PEX3*-KO (*PEX3*$^{-/-}$) AG3 cells were injected to female NOD/SCID mice at 1,000,000 cells/mouse; 1205Lu-VCDR cells were injected to male NOD/SCID mice at 2,000,000 cells/mouse.

Tumor initiation was determined once palpable tumors were formed. Tumors were then measured in length (L) and width (W). Tumor volumes (V) were calculated based on the formula $V=3.1416/6*L*W^2$. For PLX4720 treatment, mice were switched to a special diet containing PLX4720 (AIN-76A rodent diet with 417mg PLX4720/kg, Research Diets, Inc), or the corresponding control chow (AIN-76A, Research Diets, Inc), when tumor volumes reached an indicated size. For mice receiving combined BRAF and MEK inhibitors, cobimetinib (cobi) was dissolved in DMSO and subsequently diluted in PEG400 (Sigma-Aldrich, P3265), TWEEN® 20 (Sigma-Aldrich, P1379) and PBS. Mice were administered cobi (5 mg/kg) every 2 days by gavage, starting the same day as the PLX4720 special diet. For other *in vivo* drug treatment, D,L-threo-PPMP (PPMP) (Abcam, ab144023) and NNC 55-0396 (NNC) (TOCRIS Bioscience, 2268) were dissolved in DMSO at concentrations of 20 mg/mL, aliquoted and frozen in -20 °C. Before each injection, PPMP was freshly diluted in 2 parts of EtOH (70%) and 1 part of 1x PBS to a final concentration of 1.25 mg/mL; NNC was freshly diluted to a final concentration of 4 mg/mL in a solution containing 20% DMSO, 10% PEG400 (Sigma-Aldrich, P3265), and 70% PBS. For the PPMP treatment cohort, mice were kept on PLX4720 chow and were co-treated with PPMP by intraperitoneal injection at 5 mg/kg every 36 hours. Treatments were initiated when 1) the initial tumor volume of PLX4720-naïve mice reaches 200 mm^3 (Figure S4G, S4J), or 2) tumors had relapsed after PLX4720 treatment (tumor volume increase $\geq$ 10%, Figure 7B), or 3) the relapsed tumors had reached a size of 1300 mm^3 after PLX4720 treatment (Figure 7D). For the NNC treatment cohort (Figure S9A), when the tumor volume reached approximately 200 mm^3, female NOD/SCID mice were switched to PLX4720 diet and simultaneously treated with NNC by intraperitoneal injection at 20 mg/kg, and/or with PPMP by intraperitoneal injection at 5 mg/kg every 48 hours. For the PLX4720-relapsed cohort (Figure S9C), mice were kept on PLX4720 diet until tumors relapse. NNC and PPMP treatments were then initiated when relapsed tumors reached a volume of approximately 400 mm^3. For Figure 9A, PLX4720+cobi and/or PPMP+NNC treatments were initiated when A375M-dervied tumors reached an average size of 700 mm^3 and all mice were sacrificed on Day 10 after treatment initiation. For the 1205Lu-VCDR (BRAF/MEKi dual-resistant) cohort (Figure S9E), male NOD/SCID mice were used and PLX4720+cobi treatment were initiated once 1205Lu-VCDR-derived tumors were palpable. When the tumor volume reached approximately 250 mm^3, PPMP+NNC treatments were initiated while mice were kept on PLX4720 diet and treated with cobi simultaneously. Unless otherwise indicated, mice bearing D4M.3a-derived melanomas were sacrificed when the tumor volume reached 1600 mm^3; mice bearing A375M-derived melanomas were sacrificed when the tumor volume reached 1000 mm^3; and mice bearing 1205Lu-VCDR-derived melanomas were sacrificed when the tumor volume reached 1500 mm^3.

Western blotting

Cells were lysed with RIPA buffer (150mmol/L Tris-HCl, pH=7, 150mmol/L NaCl, 1% NP-40, 1% sodium deoxycholate, 0.1% SDS) supplemented with protease and phosphatase inhibitors (Roche). Equal amounts of protein samples were loaded, separated on 10% SDS-PAGE gels, transferred to PVDF membranes, and probed with corresponding antibodies listed in Table S3.

Quantitative real-time PCR

Cultured cells were pelleted, and RNA was prepared using the E.Z.N.A. total RNA isolation kit (OMEGA Bio-Tek). RNA concentrations were then quantified using a NanoDrop spectrophotometer (ThermoFisher Scientific) and cDNA was prepared from 1mg of total RNA using iScript cDNA Synthesis Kit (Bio-Rad). Target genes were quantified using the Applied Biosystems 7500 Fast Real-Time PCR System with SYBR Green real-time PCR master mix (Applied Biosystems). Two housekeeping genes were used for each assay. Primers used for qPCR are listed in Table S4.

Transmission electron microscopy (TEM)

A375M cells were allowed to grow for 48 hours following siRNA transfection to reach a confluence of 80%. D4M.3a Cas9-Ctrl (*Pex3*$^{+/+}$)*, Pex3*$^{+/-}$ Clones 6D and 9G cells were cultured to 70-80% confluence. Cells were then trypsinized and resuspended in twice the volume of culture media. Cells were initially centrifuged in 15 mL conical centrifuge tubes, and approximately 3 x10^6 cells per cell line were washed in 1x PBS, then resuspended, and transferred to 1.5 mL microcentrifuge tubes. Cells were then centrifuged at 100g for 10 minutes, washed twice in 1 mL 1x PBS, followed by aspiration of PBS, retaining cell pellets. Using 2% glutaraldehyde fixation buffer (made by mixing 0.4ml glutaraldehyde 50%, 5ml 0.2M cacodylate buffer pH7.2 and 4.6mL distilled water), pellets were fixed for one hour at 4°C. Following fixation, pellets were centrifuged at 100g for 5 minutes, and washed twice with wash/storage buffer (made by mixing equal parts of 0.2M cacodylate buffer pH7.2 with distilled water). Pellets were then processed and imaged according to previously published methods (1).

Cellular and mitochondrial bioenergetics measurements

Bioenergetic profiling was performed according to Agilent Seahorse XF Cell Mito Stress Test Kit Guidebook. Seahorse XF-96 cell culture microplates were first coated with a 50µg/ml working stock of poly-D-lysine and incubated at room temperature for 1-2 hours. 200µL of sterile water per well was added and aspirated from each well, then plates were left to air dry in an incubator without the plate lid. 20,000 A375M cells were transfected with *siCtrl* or *siPEX3* and seeded in each well overnight. The next day, cells were changed to DMSO- or vemu-containing media, then transferred to a 37°C cell incubator with 20% O_2 and 5% CO_2 for approximately 18h. Cells were then washed twice in 1x PBS and incubated with Seahorse XF medium (120µL), and incubated for 1h in a non-CO_2 37 °C incubator. XF96 sensor cartridges were placed on top of each well and 20µL, 22µL, and 25µL of oligomycin (2.5µM final), Carbonyl cyanide-4 (trifluoromethoxy) phenylhydrazone FCCP (2.0µM final), and rotenone/antimycin A (0.5µM final), were added to ports A, B, and C respectively. Plates were loaded and analyzed for cellular and mitochondrial bioenergetics according to the guidebook, alongside normalization to cell number.

Targeted Liquid chromatography tandem mass spectrometry (LC-MS-MS)

Following indicated treatments, cells were scrapped and washed twice with cold 1x PBS. Each sample was then separated into two parts: 90% cells were collected in a borosilicate tube for LC-MS-MS analysis to assess ceramide and HexCer concentrations, while 10% cells were collected in an Eppendorf tube for Bradford assay to assess total protein concentration for final normalization.

For sphingolipid extraction, exogenous odd-chain lipids (C17-ceramide (d18:1/17:0) and C17-glucosylceramide (d18:1/17:0)) were used as internal standards to quantify ceramide and HexCer species, respectively. For each sample, 25μL of the internal standard mix was added to the borosilicate tube. Then, 2mL of ice-cold chloroform:methanol (1:2) containing 0.1% Trifluoroacetic acid was added and mixed for 30 seconds by vortex. Then, liquid-liquid extraction was performed by adding 1mL of chloroform and 2mL of H_2O sequentially. The samples were mixed by vortex and centrifuged at 3,000 RPM for 10 min at 4°C to separate the aqueous (upper) and organic (lower) phases. The lower organic phase was carefully transferred into a separate test tube. The remaining material underwent a second round of extraction with an additional 1mL of chloroform. Organic phases were combined and dried under ultra-pure nitrogen gas. The final material was solubilised with 125μL of reconstitution solution (chloroform:methanol 1:9). Ceramide and HexCer species were subsequently analyzed by LC-MS-MS using Acquity Premier UPLC I-Class with Xevo TQ-S micro-System (Waters, MA, USA). Data were acquired by MassLynx software and quantified by TargetLynx software. Lipid concentrations were normalized to protein quantity, and relative lipid concentrations were presented as nmol lipid per mg protein.

Access and re-analysis of the *Banerjee 2021* yeast two-hybrid drug screen data

Raw data of the *Banerjee 2021* LOPAC yeast two-hybrid drug screen (OD_{600} reading) were retrieved from Supplementary Table 1 of the original publication (2). Figure S8A was generated in the same manner as Figure 2 in the original publication using the mean OD_{600} data of two independent screens (2). Percent growth inhibition was then calculated as 100-100*($OD_{600:\ compound}$/$OD_{600:\ DMSO}$). To assess the activity of each compound against human PEX3-PEX19 binding, the percent inhibition of the BD-PEX3/AD-PEX19-expressing yeast (%Inhibition $_{PEX3\text{-}PEX19}$) was compared to the percent inhibition of the pGAD424/pGBT9-expressing yeast (%Inhibition $_{general\ growth}$) grown in the presence of the same compound. The relative inhibition of human PEX3-PEX19 binding (Figure S8B, Y axis) was calculated as %Inhibition $_{PEX3\text{-}PEX19}$ - %Inhibition $_{general\ growth}$.

PEX3 docking

Docking studies were performed with PEX3 and NNC using Autodock Vina (3) based on the crystal structures of enzymes as deposited in the RCSB Protein Data Bank (https://www.rcsb.org/). NNC was centered at the location of PEX19-binding site on PEX3, and genetic algorithm runs were performed for the ligand and receptor.

Colony formation assay

MAPKi-resistant melanoma cells were seeded into 6-well plates at 100,000 cells per well in the presence of indicated MAPK inhibitors. The next day, NNC (4μM), PPMP (see Table S1), or DMSO control were added to the

indicated wells. Cells were subsequently cultured for 5 days, during which media was changed and drugs were freshly added on Day 3. At the end of the assay, cells were fixed with 4% formaldehyde/PBS, stained with 0.5% of crystal violet (Sigma-Aldrich, HT90132) diluted in 70% EtOH and photographed.

CIBERSORTx

CIBERSORTx was run as described (4) through https://cibersortx.stanford.edu/index.php. Briefly, *Rambow 2018* scRNA-seq data was used to generate the SMC Signature Matrix file (Table S7). Relative SMC fractions in each patient sample were then calculated based on the Signature Matrix file (see Table S6).

Supplemental References

1. Chang CH, Bijian K, Wernic D, Su J, da Silva SD, Yu H, et al. A novel orally available seleno-purine molecule suppresses triple-negative breast cancer cell proliferation and progression to metastasis by inducing cytostatic autophagy. *Autophagy.* 2019;15(8):1376-90.
2. Banerjee H, LaPointe P, Eitzen G, and Rachubinski RA. A Small Molecule Inhibitor of Pex3-Pex19 Interaction Disrupts Glycosome Biogenesis and Causes Lethality in Trypanosoma brucei. *Front Cell Dev Biol.* 2021;9:703603.
3. Trott O, and Olson AJ. AutoDock Vina: improving the speed and accuracy of docking with a new scoring function, efficient optimization, and multithreading. *J Comput Chem.* 2010;31(2):455-61.
4. Newman AM, Steen CB, Liu CL, Gentles AJ, Chaudhuri AA, Scherer F, et al. Determining cell type abundance and expression from bulk tissues with digital cytometry. *Nat Biotechnol.* 2019;37(7):773-82.
